# Derepression of Y-linked multicopy protamine-like genes interferes with sperm nuclear compaction in *D. melanogaster*

**DOI:** 10.1073/pnas.2220576120

**Published:** 2023-04-10

**Authors:** Jun I. Park, George W. Bell, Yukiko M. Yamashita

**Affiliations:** ^a^Life Sciences Institute, University of Michigan, Ann Arbor, MI 48109; ^b^Department of Cell and Developmental Biology, University of Michigan Medical School, Ann Arbor, MI 48109; ^c^Medical Scientist Training Program, University of Michigan Medical School, Ann Arbor, MI 48109; ^d^Whitehead Institute for Biomedical Research, Cambridge, MA 02142; ^e^Department of Biology, School of Science, Massachusetts Institute of Technology, Cambridge, MA 02142; ^f^HHMI, Cambridge, MA 02142

**Keywords:** protamine, spermatogenesis, *Drosophila*

## Abstract

Across species, sperm maturation involves the dramatic reconfiguration of chromatin into highly compact nuclei that enhance hydrodynamic ability and ensure paternal genomic integrity. This process is mediated by the replacement of histones by sperm nuclear basic proteins, also referred to as protamines. In humans, a carefully balanced dosage between two known protamine genes is required for optimal fertility. However, it remains unknown how their proper balance is regulated and how defects in balance may lead to compromised fertility. Here, we show that a nucleolar protein, *modulo*, a homolog of *nucleolin*, mediates the histone-to-protamine transition during *Drosophila* spermatogenesis. We find that *modulo* mutants display nuclear compaction defects during late spermatogenesis due to decreased expression of autosomal protamine genes (including *Mst77F*) and derepression of Y-linked multicopy *Mst77F* homologs (*Mst77Y*), leading to the mutant’s known sterility. Overexpression of *Mst77Y* in a wild-type background is sufficient to cause nuclear compaction defects, similar to *modulo* mutant, indicating that Mst77Y is a dominant-negative variant interfering with the process of histone-to-protamine transition. Interestingly, ectopic overexpression of *Mst77Y* caused decompaction of X-bearing spermatids nuclei more frequently than Y-bearing spermatid nuclei, although this did not greatly affect the sex ratio of offspring. We further show that *modulo* regulates these protamine genes at the step of transcript polyadenylation. We conclude that the regulation of protamines mediated by *modulo*, ensuring the expression of functional ones while repressing dominant-negative ones, is critical for male fertility.

## Significance

Protamines are small, highly positively charged proteins that are required for packaging DNA to produce mature sperm with highly condensed nuclei capable of fertilization. Even small changes in the dosage of protamines in humans is associated with infertility. Our work reveals the presence of dominant-negative protamine genes on the Y chromosome of *Drosophila melanogaster* and shows that the precise expression of functional protamines and repression of dominant-negative protamines is a critical process to ensure male fertility.

In many species, spermatids undergo the process of nuclear compaction, an essential process to produce sperm that are capable of fertilization (1–3). Nuclear compaction is critical for the sperm’s hydrodynamic performance and protecting the paternal genome against mutagens (4–6). Nuclear compaction involves the dramatic chromatin reorganization mediated by the histone-to-protamine transition (1–5, 7, 8). Sperm nuclear basic proteins, also referred to as protamines, are small, positively charged proteins that replace histone-based nucleosomes to achieve the extreme degree of DNA compaction often seen in sperm (2). As such, these protamines are required for fertility across many different species (4).

Although protamines are essential for fertility, they are rapidly evolving across species (4, 9, 10), where the primary sequence, the number, and the functionality of protamine genes are not well conserved. For example, human and mouse protamine genes, *PRM1* and *PRM2,* are required for fertility (4, 6, 7), while *PRM2* has become nonfunctional in bulls and boars (4, 11). Closely related *Drosophila* species have independently evolved many different protamine-like genes (10): *Drosophila melanogaster* has *Mst35Ba* and *Mst35Bb* (also known as *ProtA* and *ProtB*), which are the most similar to mammalian *PRM1* and *PRM2* (3, 12, 13), as well as *Mst77F*, *Prtl99C*, and Y-linked multicopy *Mst77Y*, with evidence that several more uncharacterized genes may also be involved (14). In contrast, in *Drosophila simulans*, there is just one orthologous copy of the *ProtA/B* gene (*Prot*) as well as one ortholog each for *Mst77F* (*GD12157*) and *Prtl99c* (*GD21472*). *D. simulans* lacks *Mst77Y* (10, 14), but have evolved their own clade-specific genes that contain large regions of protamine sequences (*Dox* family genes), which are not present in *D. melanogaster* (15, 16). Surprisingly, while *ProtA* and *ProtB* are most similar to their mammalian counterparts, they are not required for fertility in *D. melanogaster* (12); instead, more divergent genes *Mst77F* and *Prtl99C* are essential (17–19). The potential function of the *D. melanogaster*-specific multicopy locus of *Mst77F* homologs (the *Mst77Y* genes) is unknown (20, 21).

Interestingly, it has been observed that mammals appear to feature their own species-specific ratios of protamine dosage (2, 11, 22, 23), and in humans, even small alterations in the ratio of *PRM1* and *PRM2* are associated with infertility (2, 23–26), suggesting that a specific balance of protamines is important for sperm DNA packaging. However, it remains unknown why carefully balanced protamine expression is important and how it is achieved to support fertility.

While studying *D. melanogaster modulo* mutants, we discovered that *modulo* transheterozygotic mutant causes misregulation of protamine genes. *modulo* mutant spermatids display decreased nuclear incorporation of protamine-like protein Mst77F and increased incorporation of its Y-linked homolog, Mst77Y, which is barely incorporated in the wild type, leading to a DNA compaction defect that explains the reported sterility of *modulo* mutant. Our data indicate that Mst77Y likely acts as a dominant-negative form of Mst77F, interfering with the process of histone-to-protamine transition. Interestingly, Mst77Y has disproportionate effects on spermatids carrying an X chromosome, leading to biased decompaction of X-bearing spermatid nuclei, although it does not lead to large effects on the sex ratio of offspring. We further find that *modulo* is involved in safeguarding polyadenylation of *Mst77F* transcript over that of the Y-linked *Mst77Y*. Our study reveals a mechanism of protamine gene expression mediated by *modulo*, balancing the correct ratio of protamine gene expression to ensure male fertility.

## Results

### *modulo* Mutant Is Defective in Sperm Nuclear Compaction.

**Fig. 1.**
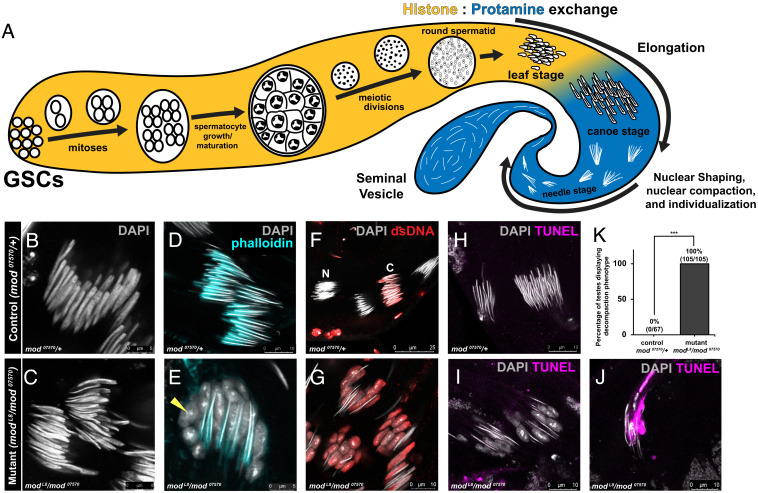
Sterility of *modulo* mutant is accompanied by defective spermatid chromatin compaction. (*A*) Schematic of spermatogenesis in *Drosophila* proceeding from germline stem cells to mature sperm. Proceeding from meiotic divisions onward, only nuclei are depicted. (*B* and *C*) Representative images of canoe-stage nuclei stained with DAPI (gray) in control males (*mod^07570^/+)* (*B*), and *modulo*-mutant males (*mod^L8^*/*mod^07570^*) (*C*). (*D* and *E*) Representative images at the stage shortly before individualization stained with DAPI (gray) and phalloidin (cyan, to visualize the individualization complex) in control males (*mod^07570^/+)* (*D*) and *modulo*-mutant males (*mod^L8^*/*mod^07570^*) (*E*). Although all nuclei eventually become decompacted in *modulo*-mutant males, individualization complex (marked by phalloidin staining) appears to be normally formed. Yellow arrowheads indicating decompacted nuclei. (*F* and *G*) Representative images of late canoe to needle stages stained with anti-dsDNA (red) and DAPI (gray) in control (*mod^07570^/+)* (*F*) and *modulo*-mutant males (*mod^L8^*/*mod^07570^*) (*G*). N: needle-stage spermatids that do not stain for dsDNA due to advanced DNA compaction, C: canoe-stage spermatids that are less compact and positive for anti-dsDNA staining. (*H*–*J*) Staining via Terminal deoxynucleotidyl transferase dUTP nick end labeling (TUNEL) (magenta) of needle-shaped spermatid cysts in control (*mod^07570^/+*) (*H*) and mutant (*mod^L8^/mod^07570^*) males without (*I*) or with (*J*) TUNEL signal. (*K*) Percentage of decompaction phenotype in *modulo*-mutant vs. wild-type males. *** indicates *P *< 0.001 (unpaired Student’s *t* test assuming unequal variances in five independent experiments). n (total number of testes counted per genotype) is presented on the bar graph.

Modulo is the *Drosophila* homolog of Nucleolin, a nucleolar protein implicated in RNA processing (27, 28). Although *modulo*-mutant males have been known to be sterile (27, 29), the cytological defects that lead to their sterility have not been characterized. We find that the *modulo* transheterozygote mutant (*mod  ^L8^*/*mod  ^07570^*) exhibits defects in nuclear morphology transformation during late spermiogenesis. In wild-type males, postmeiotic spermatid nuclei undergo well-documented morphological changes (1), from round spermatid stage, to “leaf” stage, to “canoe” stage, resulting in highly condensed “needle-” stage nuclei, which is accompanied by the histone-to-protamine transition (Fig. 1*A*). Although *modulo*-mutant germ cells proceeded through spermatogenesis normally, including early nuclear compaction (Fig. 1 *B* and *C*), the *modulo* mutant exhibited striking “decompaction” of the nuclei after reaching the canoe stage, coinciding with the individualization of spermatids (Fig. 1 *D* and *E*). Immunofluorescence (IF) staining using anti-dsDNA, which has been previously used to assess the compaction status of spermatid nuclei (30), revealed that defective spermatid nuclei of *modulo* mutant are indeed decompacted (Fig. 1 *F* and *G*). Decompacting nuclei are initially negative via Terminal deoxynucleotidyl transferase dUTP nick end labeling (TUNEL), a method used to identify DNA breaks that occur during apoptosis (Fig. 1 *H* and *I*), then become TUNEL positive (Fig. 1*J*), suggesting that decompaction is not the result of cell death, but may rather be a cause. Overall, 100% of the *modulo*-mutant testes exhibited a nuclear decompaction phenotype (Fig. 1*K*), and it appeared that all nuclei eventually become decompacted and die, filling the distal end of the testis with cellular debris (*SI Appendix*, Fig. S1 *A* and *B*). The eventual death of all sperm nuclei likely results in the entire lack of mature sperm in the seminal vesicles (*SI Appendix*, Fig. S1 *C* and *D*) and the *modulo* mutant’s known sterility.

### *modulo* Mutant Fails in Histone-to-Protamine Transition.

**Fig. 2.**
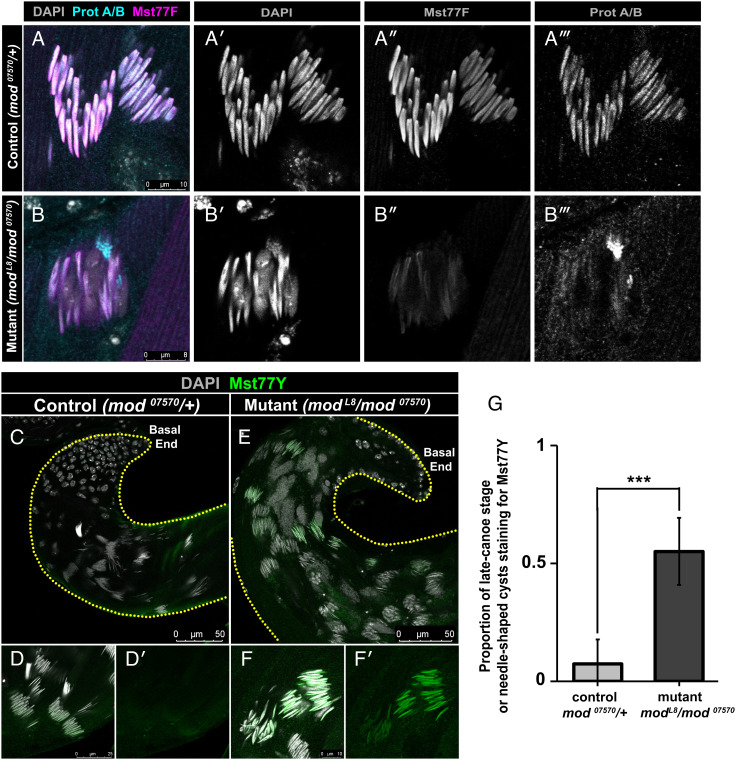
Nuclear decompaction in *modulo-*mutant spermatids is associated with decreased incorporation of Mst77F and increased incorporation of Mst77Y. (*A* and *B*) Representative images of late canoe-stage nuclei stained with DAPI (gray), anti-Prot A/B (cyan), and anti-Mst77F (magenta) in control (*mod^07570^*/+) (*A*) and mutant (*mod^L8^*/*mod^07570^*) (*B*) males. Split channel view of DAPI (*A'* and *B'*), anti-Mst77F (*A''* and *B''*), and anti-Prot A/B (*A'''* and *B'''*) in control (*A*) and mutant (*B*) males. (*C*–*F*) Representative images of canoe-stage and needle-stage spermatids at the basal end of testis stained with DAPI (gray) and anti-Mst77Y (green) in control (*mod^07570^*/+) (*C* and *D*) and mutant (*mod^L8^*/*mod^07570^*) (*E* and *F*) males. Split channel view of anti-Mst77Y in control (*D'*) and mutant (*F'*) males. Dotted lines outline the testis. (*G*) Proportion of canoe-stage cysts with nuclei positive for Mst77Y staining in mutant (*mod^L8^*/*mod^07570^*) vs. control (*mod^07570^*/+) males. *** indicates *P* ≤ 0.001 (unpaired Student’s *t* test assuming unequal variances) with n=10 testes in control and n=9 testes in mutant males from 2 independent experiments. Exact *P*-values are listed *SI Appendix*, Table S1.

Because nuclear decompaction in the *modulo* mutant occurs at stages when sperm chromatin is known to undergo reorganization through the histone-to-protamine transition, we explored whether the *modulo* mutant is defective in this process. Histone-to-protamine transition occurs step wise: 1) histone modification and removal, 2) transition protein incorporation then removal, and 3) protamine incorporation (1). IF staining revealed that *modulo*-mutant spermatids undergo proper histone removal and transient transition protein incorporation (*SI Appendix*, Fig. S2 *A*–*F*), but fail to properly accumulate ProtA/B and Mst77F (Fig. 2 *A* and *B*). Moreover, using a specific antibody (*SI Appendix*, Fig. S3 *A* and *B*), we found that Mst77Y, Y-linked multicopy homologs of Mst77F (20, 21) (*SI Appendix*, Fig. S4*A*), strongly accumulated in *modulo-*mutant spermatid nuclei, whereas it was barely detectable in control (Fig. 2 *C*–*G*), suggesting that Mst77Y is aberrantly expressed in the *modulo* mutant. As the deletion of *Mst77F* and *ProtA/B* does not cause nuclear decompaction as severe as that of the *modulo* mutant (18), we infer that the incorporation of Mst77Y (in addition to the depletion of Mst77F and ProtA/B) causes the observed catastrophic nuclear decompaction seen in the *modulo*-mutant spermatids.

### Ectopic Expression of *Mst77Y* Alone Is Sufficient to Cause Nuclear Decompaction.

The *Mst77Y* genes have several interesting features. First, the gene locus contains 18 copies of *Mst77F* homolog (*SI Appendix*, Fig. S4 *A* and *B*), which originated from a single event of *Mst77F* translocation to the Y chromosome, followed by gene amplification (20, 21). Second, many of the *Mst77Y* genes have mutations, which have resulted in changes in the position and number of critical arginine, lysine, and cysteine residues believed to be important for protamine function (4, 31). Other mutations have resulted in premature truncations (*SI Appendix*, Fig. S4*B*) (21). Note that anti-Mst77Y antibody was generated by using multiple peptides from Mst77Y that are distinct from Mst77F to increase specificity. The antibodies were also designed to be able to identify all copies of Mst77Y, which feature similar mutations and were tested to be able to identify both full-length Mst77Y (*Mst77Y-12*) and normally truncated Mst77Y (*Mst77Y-3*) (*SI Appendix*, Figs. S4 *B* and S5 *A*–*C*). Because *Mst77Y’s* mutations likely alter *Mst77F’s* normal function, we hypothesized that *Mst77Y* genes may function as a dominant-negative form of *Mst77F*. Accordingly, Mst77Y’s aberrantly high expression in the *modulo* mutant may interfere with the process of normal histone-to-protamine transition.

**Fig. 3.**
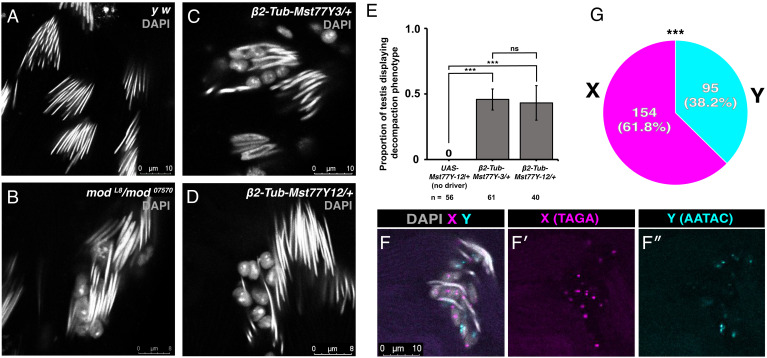
Mst77Y overexpression is sufficient to cause nuclear decompaction and causes biased decompaction of X chromosome-bearing spermatids. (*A*–*D*) Representative images of needle-stage nuclei stained with DAPI (gray) showing normal morphology in control (*y w*) (*A*), decompaction phenotype in *modulo-*mutant males (*mod^L8^/mod^07570^*) (*B*), transgenic males expressing Mst77Y-3 (truncated copy) (*C*) or Mst77Y-12 (full-length copy) (*D*) driven by *β2-tubulin* promoter. IF confirming overexpression shown in *SI Appendix*, Fig. S5 *A*–*C*. (*E*) Proportion of testes displaying decompaction phenotype in transgenic Mst77Y males. Control (*UAS–Mst77Y-12*/+) does not express Mst77Y-12 due to the absence of driver. *** indicates *P* ≤ 0.001 (unpaired Student’s *t* test assuming unequal variance), ns indicates *P* > 0.05, n = 56 testes in control, n = 61 testes in *β2-tub–Mst77Y-3/+* condition, n = 40 in *β2-tub–Mst77Y-12/+* condition from three independent experiments. (*F*) Representative images of DNA Fluorescence in situ hybridization of decompacted spermatids in Mst77Y-3-expressing males using TAGA-Cy3 (magenta, X-specific probe) (*F'*) and AATAC-Cy5 (cyan, Y-specific probe) (*F''*). (*G*) Percentage of decompacted haploid nuclei containing X chromosome vs. Y chromosome in Mst77Y-3-expressing males. *** indicates *P*(X ≥ 154) < 0.001 (exact binomial distribution) assuming *P* = 0.5 with n = 249 nuclei counted from three independent experiments. Exact *P*-values listed in Table S1.

To test the possibility that the expression of *Mst77Y* causes the nuclear decompaction phenotype, we generated transgenic lines that express *Mst77Y* under a male germline-specific *tubulin* promoter (*β2-tubulin* promoter) (32–34). From the 18 copies of *Mst77Y* homologs present on the Y chromosome (20, 21) we generated two lines expressing either *Mst77Y-12* (a full-length version) or *Mst77Y-3* (a truncated version due to premature stop codon) (*SI Appendix*, Figs. S4*B* and S6), as the transcripts of these two genes have been previously detected by qRT-PCR (21). Strikingly, expression of either *Mst77Y-3* or *Mst77Y-12* recapitulated a nuclear decompaction phenotype similar to that seen in *modulo* mutant (Fig. 3 *A*–*D*): 45.7% and 43.2% of testes examined exhibited nuclear decompaction upon expression of *Mst77Y-3* or *Mst77Y-12*, respectively (Fig. 3*E*), suggesting that high *Mst77Y* expression is sufficient to cause nuclear decompaction in a subset of spermatids. Notably, in contrast to the eventual decompaction of all spermatids seen in the *modulo* mutant, *Mst77Y* overexpression alone does not cause sterility. We speculate that this might be due to the added effect of the decreased incorporation of Mst77F and ProtA/B, in addition to high Mst77Y incorporation, seen in the *modulo* mutant.

Given that Barckmann et al. utilized the same promoter to overexpress autosomal *Mst77F* and did not observe such nuclear compaction defects during spermiogenesis (32) as we observed with *Mst77Y* overexpression, we infer that *Mst77Y* may act as a dominant-negative form, perhaps interfering with the function of *Mst77F* (*Discussion*). This notion is further supported by the fact that a truncated version (*Mst77Y-3*) also causes the decompaction phenotype. Indeed, spermatid cysts of transgenic males expressing *Mst77Y-3* exhibited uneven Mst77F staining, suggesting that some nuclei fail to accomplish proper Mst77F incorporation (*SI Appendix*, Fig. S5 *D* and *E*). It is important to note that the nuclear decompaction was most prominently observed when males were raised in 25 °C after their parents were raised at 18 °C (*Methods*). Interestingly, using DNA Fluorescence in situ hybridization (FISH) to distinguish X- vs. Y-bearing spermatids, we found that overexpression of *Mst77Y* results in biased demise of X-bearing spermatids, where 61.8% of decompacting nuclei were X-bearing, compared to only 38.2% being Y-bearing (Fig. 3 *F* and *G*). It is important to note that this bias is not due to differential efficiency of hybridization of X chromosome vs. Y chromosome DNA FISH probes: Leaf to canoe stage spermatids of control males (*SI Appendix*, Fig. S7 *A* and *B*), as well as leaf to canoe stage spermatids of *modulo*-mutant males (before they exhibit decompaction defects), exhibited ~50:50 ratio of X:Y signal (*SI Appendix*, Fig. S7 *C* and *D*), further suggesting that decompaction is biased toward X-bearing spermatids. However, a fertility assay revealed only a minor increase in the male progeny compared to sex chromosome–matched controls (51.8% vs. 47.8%, *P* = 0.0005) (*SI Appendix*, Fig. S8*A*). Likewise, only a small degree of sex ratio distortion was observed in *modulo* heterozygous mutant, compared to sex chromosome–matched control (*SI Appendix*, Fig. S8*B*) (*Discussion*).

Together, these results suggest that *Mst77Y* acts as a dominant-negative form of *Mst77F*, interfering with the incorporation of normal protamines and leading to spermatid nuclear decompaction.

### Modulo Promotes Polyadenylation of Autosomal *Mst77F* Transcript.

**Fig. 4.**
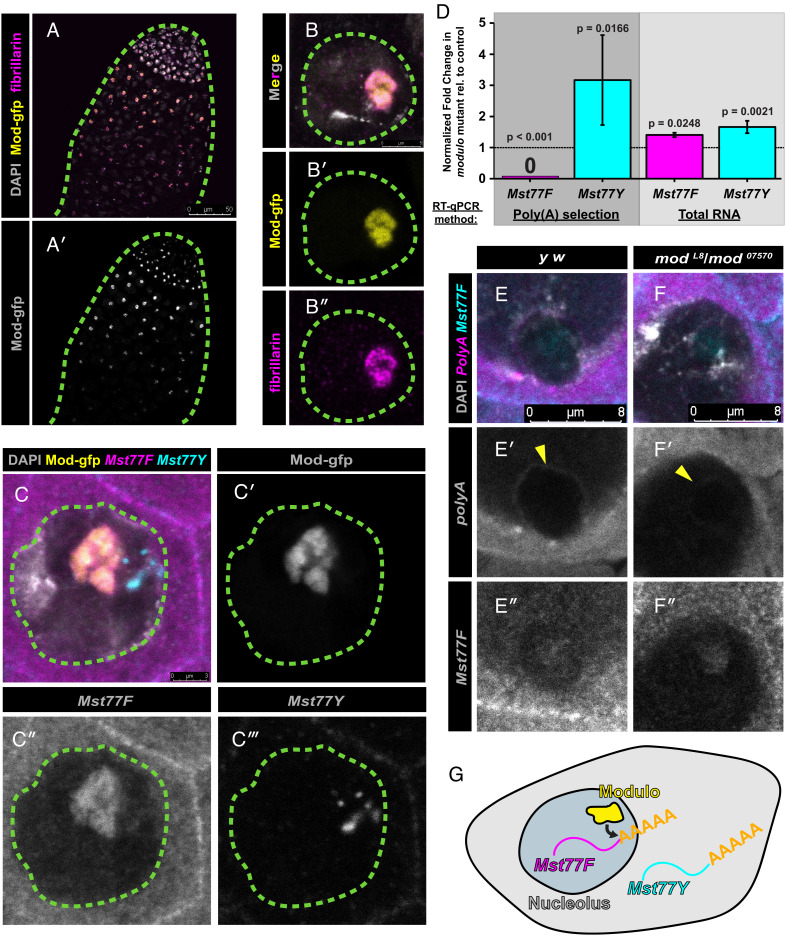
Modulo localizes to the nucleolus and functions to promote polyadenylation of *Mst77F*. (*A* and *B*) Localization of Modulo to the nucleolus in the apical tip of the testis (*A*) and in the spermatocyte nuclei (*B*). Males expressing Modulotagged with Green fluorescent protein (gfp) at the C-terminus (yellow) stained with anti-fibrillarin (magenta), a nucleolar marker, and DAPI (gray). Dotted lines outline the testis (*A*) and nucleus (*B*). (*C*) RNA FISH for *Mst77F* and *Mst77Y* transcripts in wild-type spermatocyte nucleus. DAPI (gray), Modulo–gfp (yellow), *Mst77F* (magenta), and *Mst77Y* (cyan). Split channel view of Modulo-gfp (*C'*), *Mst77F* transcript (*C''*), and *Mst77Y* transcript (*C'''*) in spermatocytes. Dotted lines outline the nucleus. (*D*) qRT-PCR following polyA selection (dark gray) or using total RNA qRT-PCR (light gray) in *modulo-*mutant males (*mod^L8^*/*mod^07570^*) vs. sibling control males *(mod*^07570^/+*)* assessing levels of *Mst77F* (magenta) and *Mst77Y* (cyan). Data were normalized to *Rp49* and control. Mean ± SD from three technical replicates is shown. *P*-values are listed (unpaired Student’s *t* test assuming unequal variance on untransformed ddct values). Similar results were obtained from two biological replicates. Primer locations are shown in *SI Appendix*, Fig. S11*A*. (*E* and *F*) RNA FISH for polyA (magenta) and *Mst77F* transcript (cyan) in control (*y w*) (*F*) vs. *modulo-*mutant males (*mod^L8^*/*mod^07570^*) (*E*), counterstained with DAPI (gray). Split channel view of polyA-containing transcripts (*E'* and *F'*) and *Mst77F* transcripts (*E''* and *F''*) in control (*E*) and mutant males (*F*). *Mst77F* probe was used to identify nucleolus. Yellow arrowhead indicates polyA-containing RNA-encircling nucleolus. (*G*) Model for Modulo function in the nucleolus.

How does *modulo* regulate the expression of *Mst77F* and *Mst77Y*? Modulo protein is expressed in the nucleolus of spermatogonia and spermatocytes, but is down-regulated prior to the meiotic divisions (Fig. 4 *A* and *B*), days earlier than the stages in which its mutant phenotype manifests. Protamine genes are known to be transcribed many days prior to the sperm nuclear compaction process in both flies and mice (3, 32, 35). Interestingly, we found that *Mst77F* transcripts colocalize with Modulo in the spermatocyte nucleolus, while *Mst77Y* transcripts localize adjacent to the nucleolus (Fig. 4*C*). These results prompted us to examine whether *Mst77F* and/or *Mst77Y* transcripts may be deregulated in *modulo* mutant. Indeed, we found that *Mst77F* messenger RNA (mRNA) is dramatically reduced in *modulo* mutant, whereas *Mst77Y* mRNA was increased approximately threefold using qRT-PCR of polyA-selected RNA (Fig. 4*D*). However, when total RNA was used for qRT-PCR or total RNA sequencing, we found that both *Mst77F* and *Mst77Y* transcripts were increased in *modulo* mutant (Fig. 4*D* and *SI Appendix*, Fig. S9*A*). RNA FISH, which visualizes total RNA, also confirmed the increase of both *Mst77F* and *Mst77Y* transcripts in *modulo* mutant (*SI Appendix*, Fig. S9*B*). Furthermore, total RNA-Seq and qRT-PCR did not detect any splicing defects of *Mst77F* or *Mst77Y* in *modulo* mutant (*SI Appendix*, Fig. S10). Collectively, these results suggest that Modulo specifically regulates transcripts of *Mst77F* and *Mst77Y* at the step of polyadenylation.

Given that Modulo protein and *Mst77F* transcript colocalize in the nucleolus, we speculate that *Mst77F* is directly regulated by Modulo, whereas increased mRNA level of *Mst77Y* may be an indirect consequence of reduced functional *Mst77F* mRNA. Interestingly, RNA FISH using poly(T) probes revealed that poly(A) signal encircles the nucleolus in wild-type spermatocytes, whereas markedly less poly(A) was detected around the nucleolus in the *modulo* mutant (Fig. 4 *E* and *F*), further suggesting that *modulo* may function to facilitate polyadenylation of transcripts localized to the nucleolus. Our findings are consistent with the known importance of polyadenylation to sperm-specific transcripts, such as protamines, which must be translationally repressed for long periods(36–39). Taken together, these results suggest that *modulo* plays an essential role in sperm nuclear compaction by facilitating maturation of canonical *Mst77F* transcript over that of Y-linked *Mst77Y* (Fig. 4*G*).

## Discussion

The present study reveals a regulatory mechanism mediated by a nucleolar protein Modulo that balances the expression of protamine subtypes in *D. melanogaster*. This finding may represent a similar theme to what is seen in the fragile balance of *PRM1* and *PRM2* in mammalian fertility (2, 7, 24, 25). In the case of *Mst77Y*, Y-linked multicopy *Mst77F* homologs, our study suggests that they have the ability to act as dominant-negative protamines and thus must be carefully regulated/repressed. The present study also confirmed that *Mst77Y* genes are expressed as proteins as suggested previously by the finding that several of the copies contain complete open-reading frames (21) and is also consistent with small RNA sequencing revealing that the *Mst77Y* locus is not a source of small RNAs (40).

We showed that overexpression of Mst77Y dominantly interferes with Mst77F incorporation, leading to decompaction of sperm nuclei and their demise. *Mst77Y* genes feature differences from their autosomal homolog that further support the idea that they are dominant-negative versions of *Mst77F* and interfere with sperm chromatin compaction. Mst77Y-12, which retains the full ORF of *Mst77F* (*SI Appendix*, Fig. S4), exhibits 87% overall sequence homology to autosomal Mst77F. At the domain/motif level, the MST-HMG-box domain, suggested to be important for DNA binding (14), maintains 100% homology, while the coiled-coil motif and C-terminal domain maintain only ~79.5% and ~85% homology, respectively (*SI Appendix*, Fig. S6*B*). It has been shown that the N-terminal domain of Mst77F, which contains the coiled-coil motif, interacts with the C-terminal domain to induce multimerization to mediate DNA compaction (41). The changes to Mst77Y at important regions may thus influence the multimerization of protamines and the formation of proper sperm chromatin structure, by interfering with the ability of the canonical version to multimerize. The notion that Mst77Y behaves as a dominant-negative version of Mst77F is further supported by the fact that overexpression of Mst77Y-3, a truncated version which does not contain the C-terminal domain (*SI Appendix*, Fig. S6*B*), is still sufficient to cause defects in nuclear compaction (Fig. 3*C*).

What is the potential “function” of dominant-negative protamines? We propose a few nonmutually exclusive possibilities. First, dominant-negative protamines may participate in meiotic drive, as suggested by recent works in *D. simulans* (15, 16) as well as *D. melanogaster* (10). Indeed, our data suggest that Mst77Y has the ability to disproportionally affect X-bearing spermatids. While this did not result in a large sex ratio distortion in offspring (*SI Appendix*, Fig. S8), this ability to harm a subset of developing spermatids during postmeiotic development may indicate the possibility that these protamine variants could be exploited by a meiotic drive system to unleash its own selfish purpose. Intriguingly, studies on the Winters sex-ratio meiotic drive system in *D. simulans* revealed that the driver, *Dox,* contains a large portion of the *Protamine* gene (15, 16). While it has not been confirmed whether this protamine-like region is essential for sex ratio distortion, the derepression of *Dox* does seem to cause nuclear defects during spermiogenesis (42). We propose that a drive system that would be able to localize a dominant-negative protamine such as Mst77Y to a subset of spermatids containing one homolog over another could be quite successful at achieving bias. Alternatively, the dominant-negative version of a protamine may be utilized when spermatogenesis needs to be aborted (similar to the concept of “programmed cell death”), for example under stress conditions. In such a case, dominant-negative protamines (such as *Mst77Y*) can be up-regulated to lead to abortive spermatogenesis. In such a scenario, a dominant-negative protamine may have a beneficial function for the organism. Yet another possibility that may contribute toward the rapid divergence of protamines is that protamine genes evolve to optimally package the genome, which may be greatly influenced by the composition of the most abundant sequences in a given genome, i.e., repetitive DNA such as satellite DNA. As these repetitive sequences are known to rapidly diverge across species (43), protamine genes may have to adapt to accommodate diverged repetitive DNA sequences, leading to rapid divergence and/or emergence of multiple protamine genes to optimally package different repetitive DNA with distinct structure/sequence. In such a scenario, fine-tuning the expression of different protamine genes may be critical. Additionally, if any protamine genes have evolved to optimally package certain satellite DNA, conversion of such protamine into a dominant-negative version can immediately target the chromosome that harbors the given satellite DNA, leading to meiotic drive that selectively harms the specific chromosome. This possibility is intriguing as the *Segregation Distorter* (*SD*) meiotic drive system in *D. melanogaster* is known to target *Responder* satellite DNA repeats (44–46) and exhibits sperm nuclei decompaction similar to what is observed in this study (30). The possibility that dominant-negative protamines are involved in the decompaction of spermatid nuclei in the *SD* drive system remains to be studied.

Taken together, our study identified a mechanism by which various protamine variants are coordinately regulated at the posttranscriptional level, possibly to achieve balanced expression of multiple protamine variants. A similar mechanism may be at play to fine-tune the expression levels of protamine variants in human and mouse, disruption of which is associated with compromised fertility.

## Methods

### Fly Husbandry and Strains.

All fly stocks were raised on standard Bloomington medium at 25 °C, and young flies (1- to 3-d-old adults) were used for all experiments unless otherwise specified. Flies used for wild-type experiments were the standard laboratory wild-type strain *y w* (y^1^w^1^). The following fly stocks were used: *modulo^07570^*/TM3 [Bloomington Drosophila Stock Center (BDSC): 11795], *modulo^L8^*/*TM3* (BDSC: 38432), and C(1)RM/C(1;Y)6, *y^1^w^1^f^1^*/0 (BDSC: 9460). The *β2-tubulin* promoter sequence used for producing *Mst77Y* overexpression was generously provided by Peiwei Chen and Alexei Aravin.

The *Mst77Y* transgenic flies were generated by phiC31 site–directed integration into the Drosophila genome. For *UAS–Mst77Y-12*, *β2-tubulin–Mst77Y-3*, and *β2-tubulin–Mst77Y-12* transgenic lines, the *Mst77Y* overexpression sequences in *D. melanogaster* were synthesized by gene synthesis service from Thermo Fisher Scientific (GeneArt Gene Synthesis) and were cloned into *pattB* vector to insert into specific integration site on second chromosome (*attP40*) (*SI Appendix*, Fig. S5*C* and Table S2). All injection and selection of flies containing integrated transgene were performed by BestGene Inc. Because *UAS–Mst77Y-12* transgene was injected to the same host fly strain as *β2-tubulin–Mst77Y-3*, and *β2-tubulin–Mst77Y-12* transgenic lines, we used this (without *gal4* driver) as a “background-matched control.”

*Modulo–gfp* strain was constructed using CRISPR-mediated knock-in of a Green fluorescent protein (gfp)-tag at the C terminus of Modulo (Beijing Fungene Biotechnology Co.) (*SI Appendix*, Table S3).

### Sex Ratio Assay.

Individual 1-d-old males raised for at least one generation at 18 °C were crossed with 3× 1- to 3-d-old virgin *y w* females at 25 °C. After 1 d, males were removed. This was done to maximize the proportion of males exhibiting decompaction phenotype described in Fig. 3. Females were left to produce embryos for a total of 5 d before cleared. Following the onset of eclosion, sex of offspring was scored for 10 consecutive days.

### RNA Fluorescent In Situ Hybridization.

All solutions used were Rnase free. Testes from 1- to 3-d-old flies were dissected in 1X phosphate buffered saline (PBS) and fixed in 4% formaldehyde in 1X PBS for 30 min. Then, the testes were washed briefly in PBS and permeabilized in 70% ethanol overnight at 4 °C. For strains expressing gfp (i.e., Modulo–gfp), the overnight permeabilization in 70% ethanol was omitted. The testes were briefly rinsed with wash buffer (2X saline-sodium citrate (SSC), 10% formamide) and then hybridized overnight at 37 °C with fluorescently labeled probes in hybridization buffer [2X SSC, 10% dextran sulfate (sigma, D8906), 1 mg/mL *E. coli* transfer RNA (sigma, R8759), 2 mM vanadyl ribonucleoside complex (NEB S142), 0.5% Bovine serum albumin (BSA) (Ambion, AM2618), 10% formamide]. Following hybridization, samples were washed two times in wash buffer for 30 min each at 37 °C and mounted in VECTASHIELD with DAPI (Vector Labs).

Fluorescently labeled probes were added to the hybridization buffer to a final concentration of 100 nM. Poly(T) probes for recognizing Poly(A) sequence were from Integrated DNA Technologies. Probes against *Mst77F* and *Mst77Y* were designed using the Stellaris1 RNA FISH Probe Designer (Biosearch Technologies, Inc.) available online at www.biosearchtech.com/stellarisdesigner. Each set of custom Stellaris1 RNA FISH probes was labeled with Quasar 670 or Quasar 570 (*SI Appendix*, Table S4).

Images were acquired using an upright Leica TCS SP8 confocal microscope with a 63× oil immersion objective lens (NA = 1.4) and processed using Adobe Photoshop and ImageJ software.

### DNA Fluorescence In Situ Hybridization.

Testes from 1- to 3-d-old flies were rapidly dissected in 4% formaldehyde and 1mM Ethylenediaminetetraacetic acid (EDTA) in 1X PBS and then nutated for 30 min. Then, the testes were washed three times in 1X PBS containing 0.1% Triton-X (PBST) +1 mM EDTA for 30 min each. The testes were briefly rinsed with 1X PBST and then incubated at 37 °C for 10 min with 2 mg/mL Rnase A in PBST. Following Rnase treatment, samples were washed once in 1X PBST + 1 mM EDTA for 10 min. The samples were then briefly rinsed with 2X SSC + 1 mM EDTA + 0.1% Tween-20, and then washed three times in 2X SSC + 0.1% Tween-20 + formamide (20% for first wash, 40% for second, 50% for third) for 15 min each. The samples were then washed with 2X SSC + 0.1% Tween-20 + 50% formamide for 30 min. The samples were then incubated for 5 min at 95 °C with fluorescently labeled probes in hybridization buffer (2X SSC, 10% dextran sulfate, 50% formamide, 1 mM EDTA) and then transferred to 37 °C overnight. Following hybridization, the samples were washed three times in 2X SSC + 1 mM EDTA + 0.1% Tween-20 for 20 min each and then mounted in VECTASHIELD with DAPI (Vector Labs).

Fluorescently labeled probes were added to the hybridization buffer to a final concentration of 500 nM. Satellite DNA probes distinguishing X and Y chromosomes (AATAC)_6_-Cy5 for Y and (TAGA)_8_-Cy3 were from Integrated DNA Technologies.

### IF Staining.

Testes were dissected in 1X PBS, transferred to 4% formaldehyde in 1X PBS, and fixed for 30 min. The testes were then washed three times in 1X PBST for 20 min each followed by incubation with primary antibodies diluted in 1X PBST with 3% BSA at 4 °C overnight. Samples were washed three times in 1X PBST for 20 min each and incubated with secondary antibody in 1X PBST with 3% BSA for 2 h at room temperature. The samples were then washed three times in 1X PBST for 20 min each and mounted in VECTASHIELD with DAPI (Vector Labs).

The following primary antibodies were used: anti-fibrillarin (1:200, mouse; Abcam; ab5812), anti-Modulo (1:1,000, guinea pig; this study), anti-protamine A/B [1:200, guinea pig, gift of Elaine Dunleavy, Centre for Chromosome Biology, National University of Ireland, Galway, Ireland (47), anti-dsDNA (1:500; mouse,; Abcam; ab27156), anti-histone H3 (1:200, rabbit; Abcam; ab1791), anti-Mst77F (1:1,000; guinea pig, this study), anti-Mst77Y (1:500; rabbit, this study), anti-Tpl94D (1:500; rabbit, this study), and phalloidin-Alexa Fluor 546 or 488 (1:200; Thermo Fisher Scientific; A22283 or A12379). The Modulo antibody was generated by injecting a peptide sequence CRKQPVKEVPQFSEED[48-62] targeting the N-terminal end of Modulo in guinea pigs (Covance). The Tpl94D antibody was generated by injecting a peptide DKGSAYKPLTLNRSYVIRKC[96-114] in rabbits (Covance). The Mst77F antibody was generated by injecting multiple peptides (SKPEVAVTC[9-16], YKKSIEYVNC[22-30], CRSSEGEHR[112-119], LQRSSEGEHRMHSEC[110-123], RSSGKPKPKGARPRKC[169-183]) targeting sites in Mst77F, as indicated, differentiating it from Mst77Y in guinea pigs (Covance). The Mst77Y antibody was generated by injecting multiple peptides (IKPDVAVSC[9-16], SRKAIEYVKC[22-30], CRSIEAELR[112-119], KTSRKAIEYVKSD[20-32], CVSSLQRSIEAELR[107-119]) targeting sites of varying aa length in Mst77Y, differentiating it from Mst77F in rabbits (Covance). Alexa Fluor–conjugated secondary antibodies (Life Technologies) were used at a dilution of 1:200.

### qRT-PCR.

Total RNA was purified from *D. melanogaster* adult testes (75 pairs/sample) by Direct-zol RNA Miniprep (Zymo Research), with DNase treatment according to manufacturer’s protocol. One microgram total RNA was reverse transcribed following priming with either random hexamers or polyT using SuperScript III® Reverse Transcriptase (Invitrogen) followed by qPCR using Power SYBR Green reagent (Applied Biosystems). Primers for qPCR were designed to amplify mRNA or intron-containing transcript as indicated. Relative expression levels were normalized to Rp49 and control siblings. All reactions were done in technical triplicates with at least two biological replicates. Graphical representation was inclusive of all replicates and *P*-values were calculated using a *t* test performed on untransformed average ddct values. Primers used are described in *SI Appendix*, Fig. S11 *A* and *B*.

### Total RNA-Seq.

Total RNA was purified from *D. melanogaster* adult testes by Direct-zol RNA Miniprep (Zymo Research), with Dnase treatment. Quality of the indexed libraries was confirmed using an Agilent Fragment Analyzer and qPCR. Sequencing libraries were prepared with the KAPA RNA HyperPrep Kit with RiboErase. Samples were sequenced on a HiSeq 2500, producing 100 × 100 nt paired-end reads. The read pairs were mapped to the canonical chromosomes of the *D. melanogaster* genome (assembly BDGP6/dm6) using STAR 2.7.1a (48)*;* default parameters, except “—alignIntronMax 25000,” indexed with all FlyBase genes (FB2020_06 Dmel Release 6.37) and the option “—sjdbOverhang 100.” Gene counts were obtained using featureCounts (49); v 2.0.1, with “-M –fraction -p -s 2.” After summing gene counts for technical replicates, differential expression was assayed using DESeq2 v1.26.0 (50), with lfcShrink(type=”ashr”)). RNA coverage across genes at nucleotide resolution was quantified with “bedtools coverage” (51) and scaled by the total number of reads mapped to genes.

### Statistics and Reproducibility.

Data are presented as mean ± SD unless otherwise indicated. The sample number (n) indicates the number of testes, nuclei, or male flies in each experiment as specified in the figure legends. We utilized two-sided Student’s *t* test to compare paired or independent samples, as applicable and is specified in the figure legends. We calculated probability using exact binomial distribution with parameters specified in Fig. 3*G* legend. No statistical methods were used to predetermine sample sizes. The experimenters were not blinded to the experimental conditions, and no randomization was performed. All the statistical details of the experiments are provided in the main text and legends. *P*-values are listed either in figure, figure legends, or *SI Appendix*, Table S1.

## Supplementary Material

Appendix 01 (PDF)

## Data Availability

Sequencing data is available at National Center for Biotechnology Information Gene Expression Omnibus under accession GSE214456 (52). All other data are included in the manuscript and/or *SI Appendix*.
