# Supplementary material for: Derepression of Y-linked multicopy protamine-like genes interferes with sperm nuclear compaction in D. melanogaster
Source: Proc Natl Acad Sci U S A. 2023 Apr 10;120(16):e2220576120. doi: 10.1073/pnas.2220576120 (PMC10120018; doi:10.1073/pnas.2220576120)
Supplement: Supplementary file 1 — Appendix 01 (PDF) [file pnas.2220576120.sapp.pdf]

Supplementary figures and tables

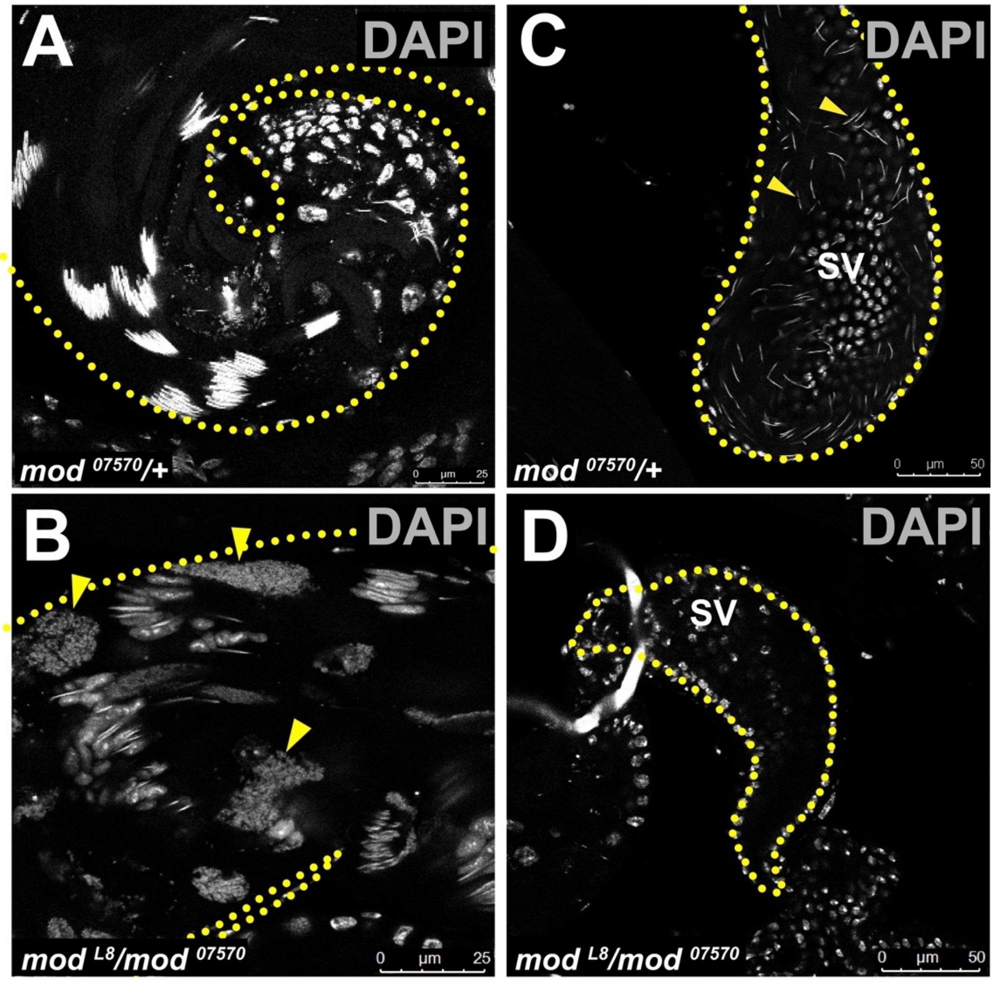

Fig. S1. *modulo* mutant results in sterility with complete spermatid demise and empty seminal vesicles.

A, B) Representative images of the testis basal end of control (mod^{07570}/+) (A) and mutant (mod^{L8}/mod^{07570}) (B) males. Arrowheads pointing to widespread DAPI-positive debris present in *modulo* mutant. DAPI (grey). Dotted line outlines the testis basal end (A, B)

C, D) Representative images of seminal vesicle of control (mod^{07570}/+) (C) and mutant (mod^{L8}/mod^{07570}) (D) male. 'SV' indicating seminal vesicle. DAPI (grey). Dotted line outlines the seminal vesicle (C, D).

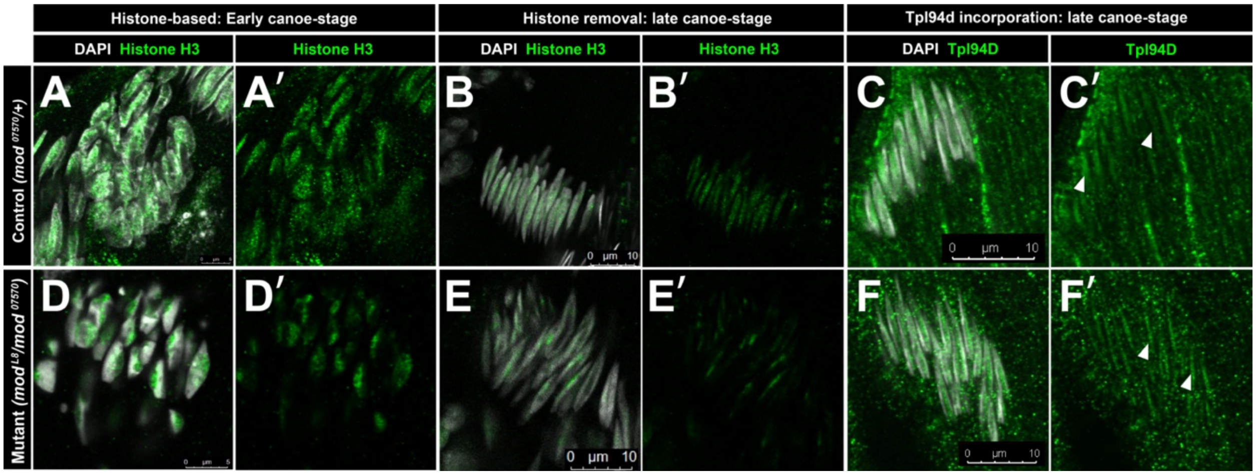

Fig. S2. *modulo* mutant proceeds normally through early stages of histone-to-protamine transition

A-F) Representative images of control (*mod*^{07570}/+) (A-C) and mutant (*mod*^{L8}/*mod*^{07570}) (D-F) spermatids undergoing appropriate histone (green) (A', B', D', E') removal and transition protein incorporation (green) (C', F').

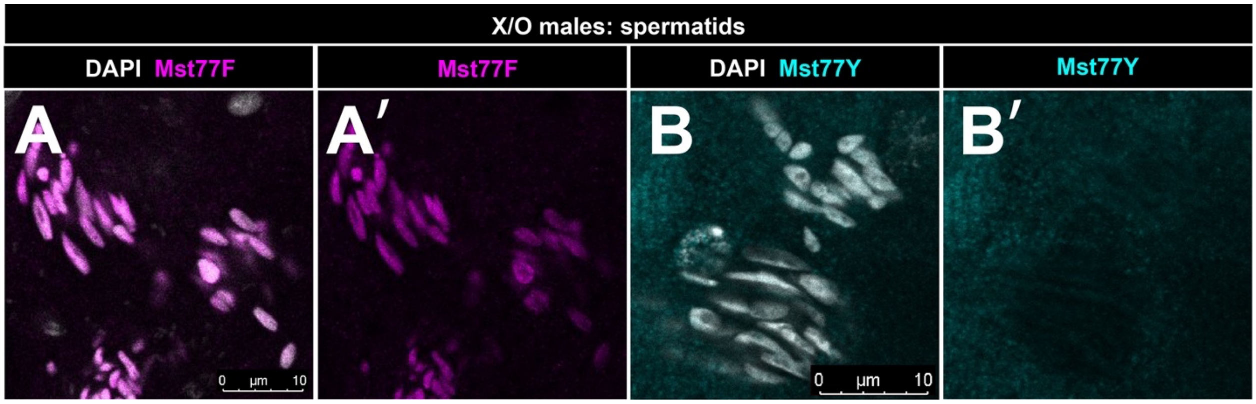

Fig. S3. Specificity of Mst77Y antibody

A) Representative image of IF using anti-Mst77F (magenta) (A') in XO males, showing expected presence of Mst77F, an autosomal gene.

B) Representative image of IF using anti-Mst77Y (cyan) (B') in XO males showing expected absence of Mst77Y, a Y-chromosome gene.

A

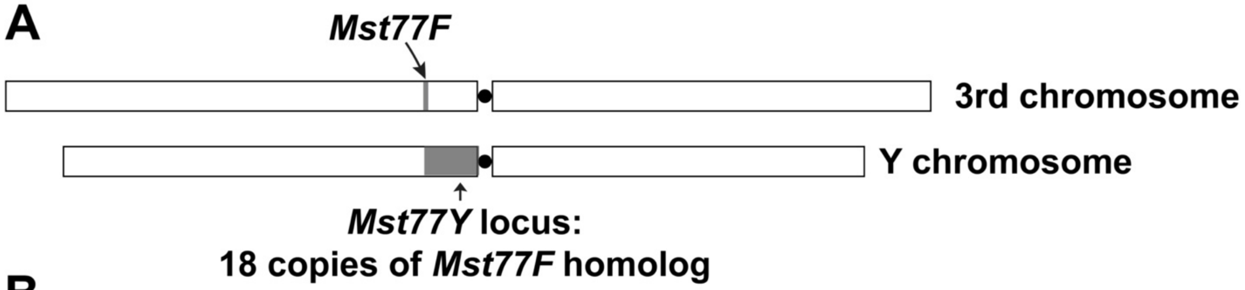

B

Compiled from Krsticevic et al. 2010, Krsticevic et al. 2015 (*20*, *21*)

ID	Estimated copy number	Expressed? (Detected via qRT-PCR)	Size (aa)	Notes
Mst77Y-1	1	yes	214	Intact ORF
Mst77Y-3	1	yes	118	Frameshift -> Premature stop codon (PSC)
Mst77Y-4	3	no	213	Intact ORF
Mst77Y-6	2	yes	71	PSC
Mst77Y-7	2	yes	214	Intact ORF
Mst77Y-10	1	yes	188	Frameshift -> PSC
Mst77Y-12	3	yes	214	Intact ORF
Mst77Y-13	1	yes	214	Intact ORF
Mst77Y-15	1	no	81	Frameshift -> PSC
Mst77Y-16	1	no	188	Frameshift -> PSC
Mst77Y-17	1	yes	188	Frameshift -> PSC
Mst77Y-18	1	no	115	Frameshift -> PSC

Fig. S4. *Mst77Y* locus contains 18 copies of *Mst77F* homolog located on the Y-chromosome

A) Location of *Mst77Y* and *Mst77F* loci. Shaded regions are not drawn to scale.

B) Summary of *Mst77Y* genes (18 copies of *Mst77F* homologs), compiled from Krsticevic et al. 2010 (*20*) and Krsticevic et al. 2015 (*21*). Yellow highlighted rows indicate the genes used in this paper for overexpression in transgenic animals.

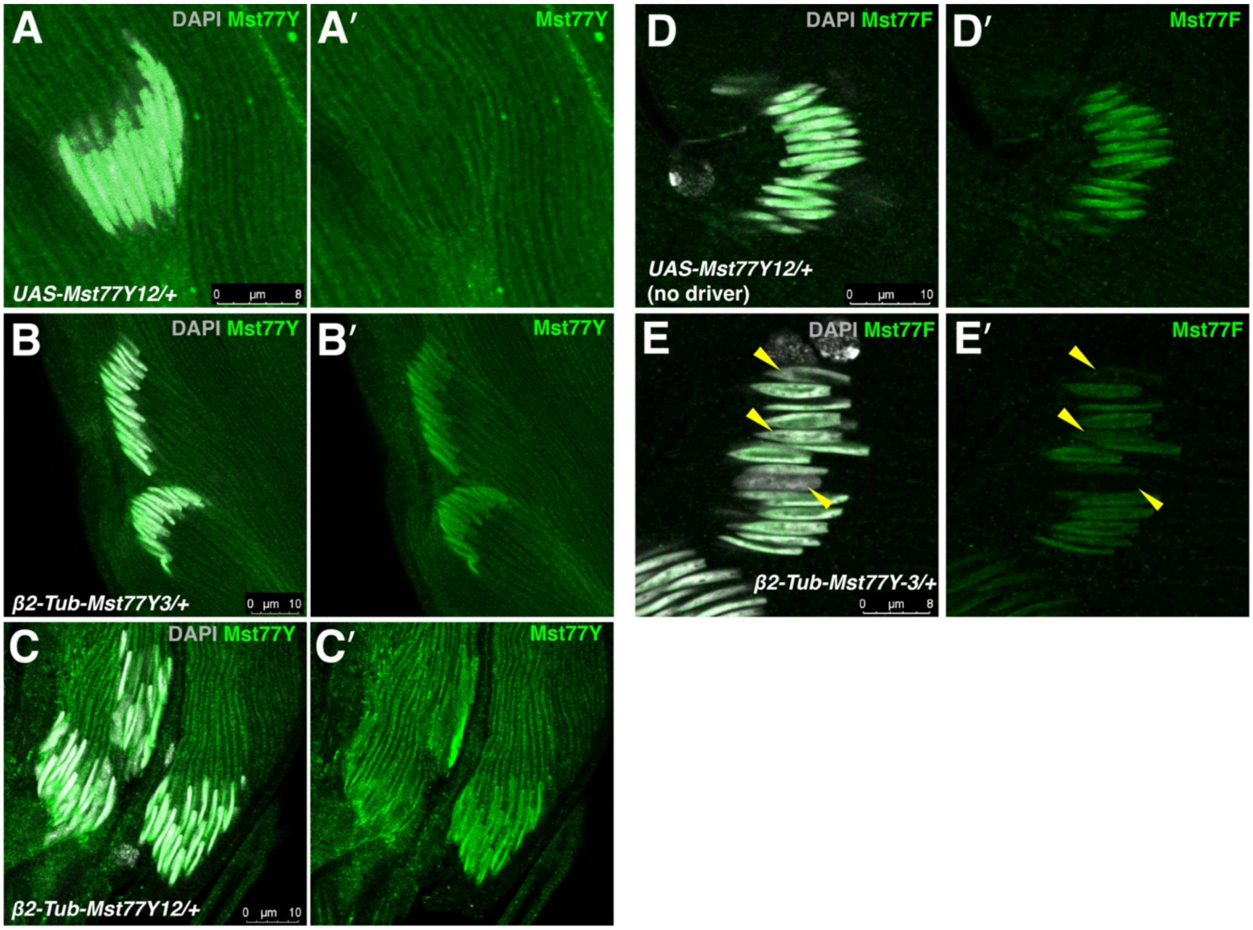

Fig. S5. Transgenic *Mst77Y* flies overexpress Mst77Y and display Mst77F incorporation defects.

A-C) Representative images of IF using anti-Mst77Y (green) (A'-C') in control (*UAS-Mst77Y-12*/+, without gal4 driver) (A), *β2-tub-Mst77Y-3*/+ (B), and *β2-tub-Mst77Y-12*/+ (C) males, counterstained with DAPI (grey).

D, E) Representative images of IF using anti-Mst77F (green) (D', E') in control (*UAS-Mst77Y-12/+*) with no driver (D) and *β2-tub-Mst77Y-3* (E) males, counterstained with DAPI (grey). Yellow arrowheads indicate spermatid nuclei within the same cyst with decreased Mst77F incorporation.

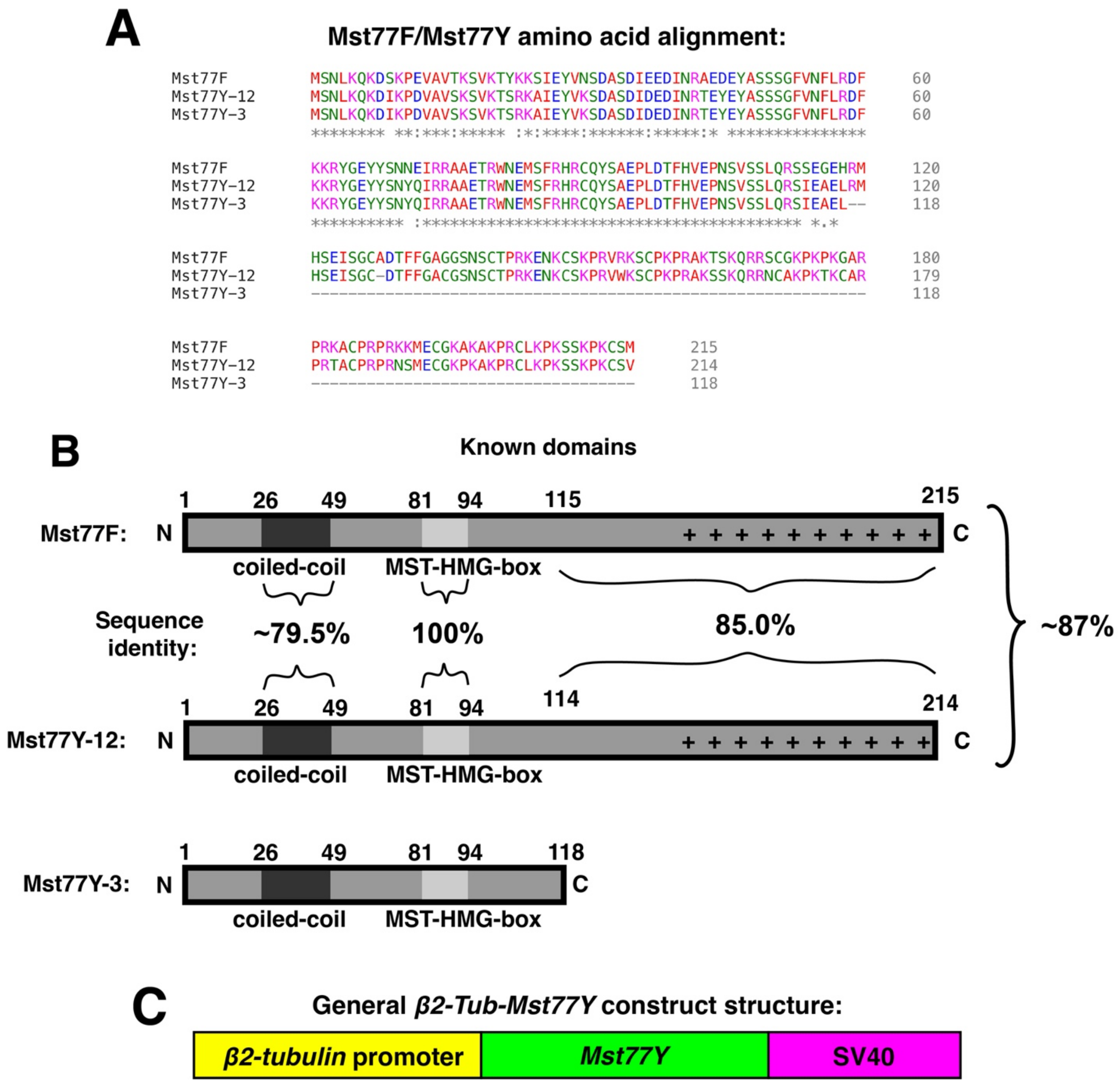

Fig. S6. Schematic of *β2-tub*-Mst77Y constructs.
A) Amino acid sequence alignment of Mst77F, Mst77Y-3, Mst77Y-12. Asterisks (*) represent identical residue, colons (:) represent non-identical but similar amino acids, blank space indicates non-identical and non-similar amino acids, and hyphens (-) indicate missing residues.
B) Schematic of previously described Mst77F domains based on Doyen et al., 2015 (***14***) and Kost et al., 2015 (***41***). "+" indicates region of highly positively charged residues.
C) Structure of transgenic construct, consisting of *β2-tubulin* promoter followed by *Mst77Y* ORF (either *Mst77Y-12 or Mst77Y-3*), stop codon and SV40 3'-UTR. Sequence information contained in Table S2.

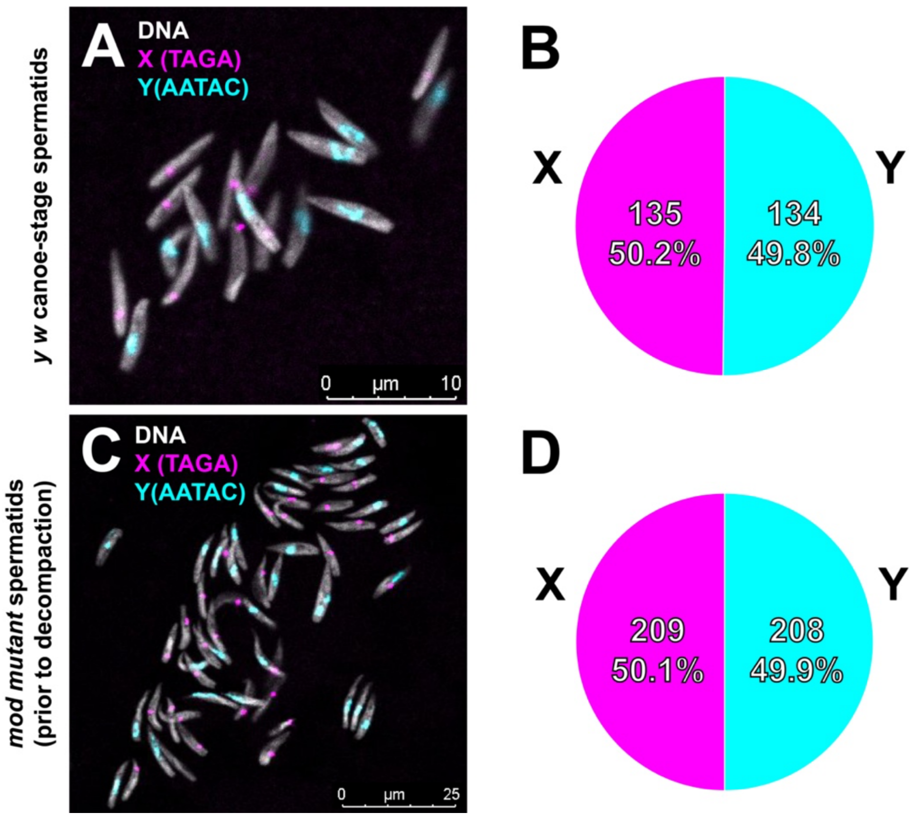

Fig. S7. Spermatids normally contain equal 50:50 ratio of X and Y chromosomes
A, C) Representative images of DNA FISH against satellite DNA sequences that are unique in X chromosome (TAGA, magenta) and Y chromosome (AATAC, cyan) in control males (*y w*) (A) and *modulo* mutant males (*mod*^{L8}/*mod*^{07570}) before exhibiting compaction defects (C).
B, D) Percentage of round-canoe stage spermatid nuclei containing X (magenta) or Y (cyan) chromosomes in control males (*y w*) (B) and *modulo* mutant males (*mod*^{L8}/*mod*^{07570}) (D). n = 269 nuclei counted from 3 independent experiments (B). n = 417 nuclei counted from 3 independent experiments (D).
These results demonstrate that DNA FISH detection of X and Y chromosomes is not artifactually biased.

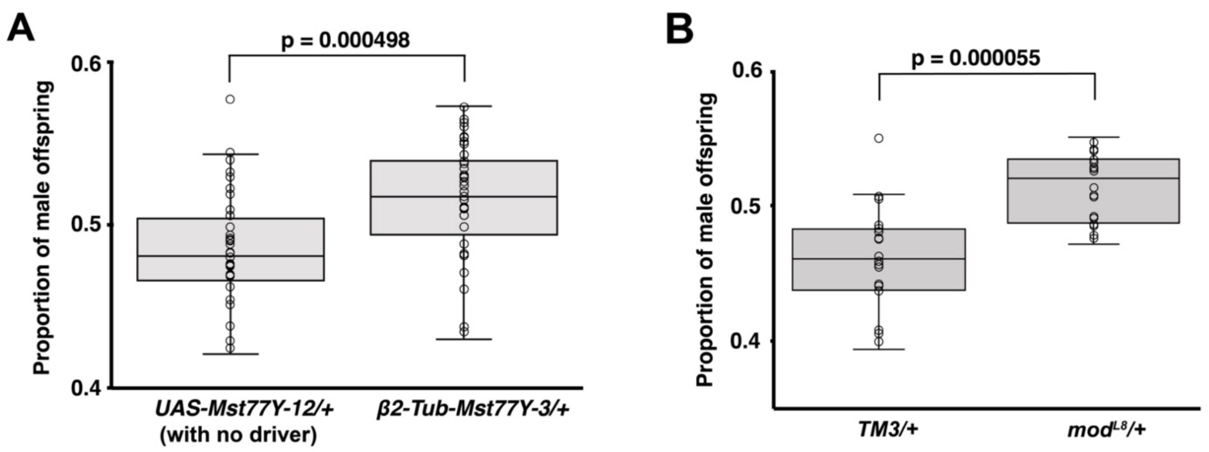

Fig. S8. *modulo* mutant heterozygote males and Mst77Y overexpressing males display male-biased sex ratio

Box and whisker plot showing proportion of male offspring produced by transgenic males in a sex ratio assay.

A) Control males (*UAS-Mst77Y-12*/+) with no driver or Mst77Y overexpressing males (*β2-tub-Mst77Y-3*/+) were crossed with 3 *y w* virgin females and sex of resultant offspring were scored. *UAS-Mst77Y-12*/+ males were used as control as they do not express Mst77Y and contains transgene inserted into same site as experimental condition. *P*-value shown in figure (unpaired Student's t-test assuming unequal variance) with n = 35 individual crosses in control and n = 36 individual crosses in experimental condition from 3 independent experiments.

B) Control males (*TM3/+)* or sibling *modulo* mutant heterozygote males (*mod*^{L8}/+) with identical sex chromosome (X/Y) background were crossed with 3 *y w* virgin females and sex of resultant offspring were scored. *P*-value shown in figure (unpaired Student's t-test assuming unequal variance) with n = 19 individual crosses in control and n = 18 individual crosses in experimental condition from 3 independent experiments.

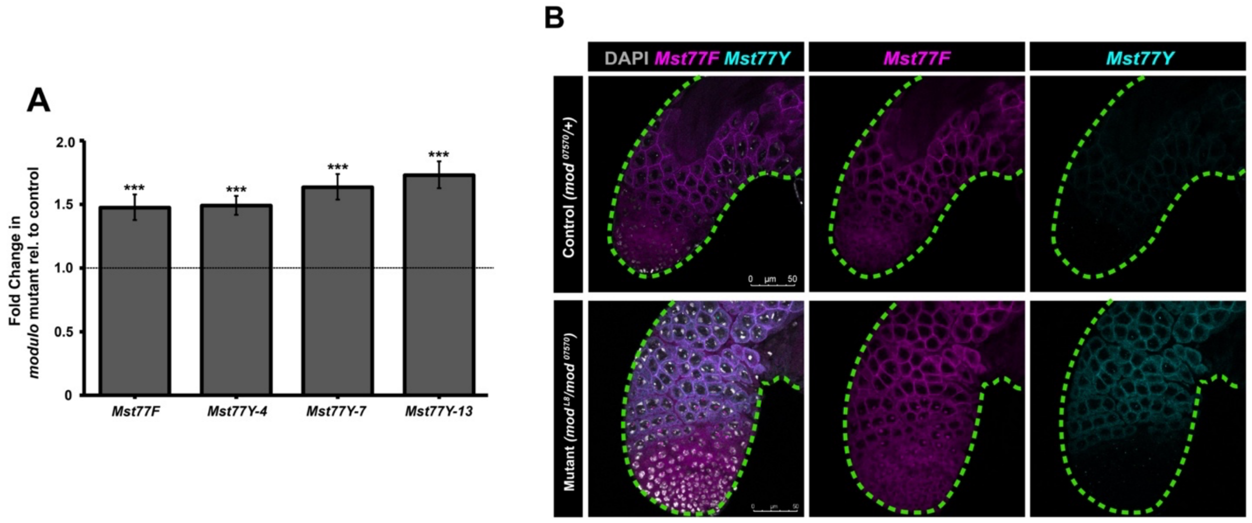

Fig. S9. *modulo* mutant results in up-regulation of *Mst77F* and *Mst77Y* total RNA.
A) Fold-change of *Mst77F* and *Mst77Y* RNA in *modulo* mutant (mod^{L8}/mod^{07570}) compared to control (mod^{07570}/+) using total RNA-seq. *** indicates $P < 0.001$ (unpaired Student's t-test assuming unequal variance) with n = 3 independent experiments.
B) Representative images of RNA FISH of *Mst77F* (magenta) and *Mst77Y* (cyan) in control (mod^{07570}/+) and *modulo* mutant (mod^{L8}/mod^{07570}) males.

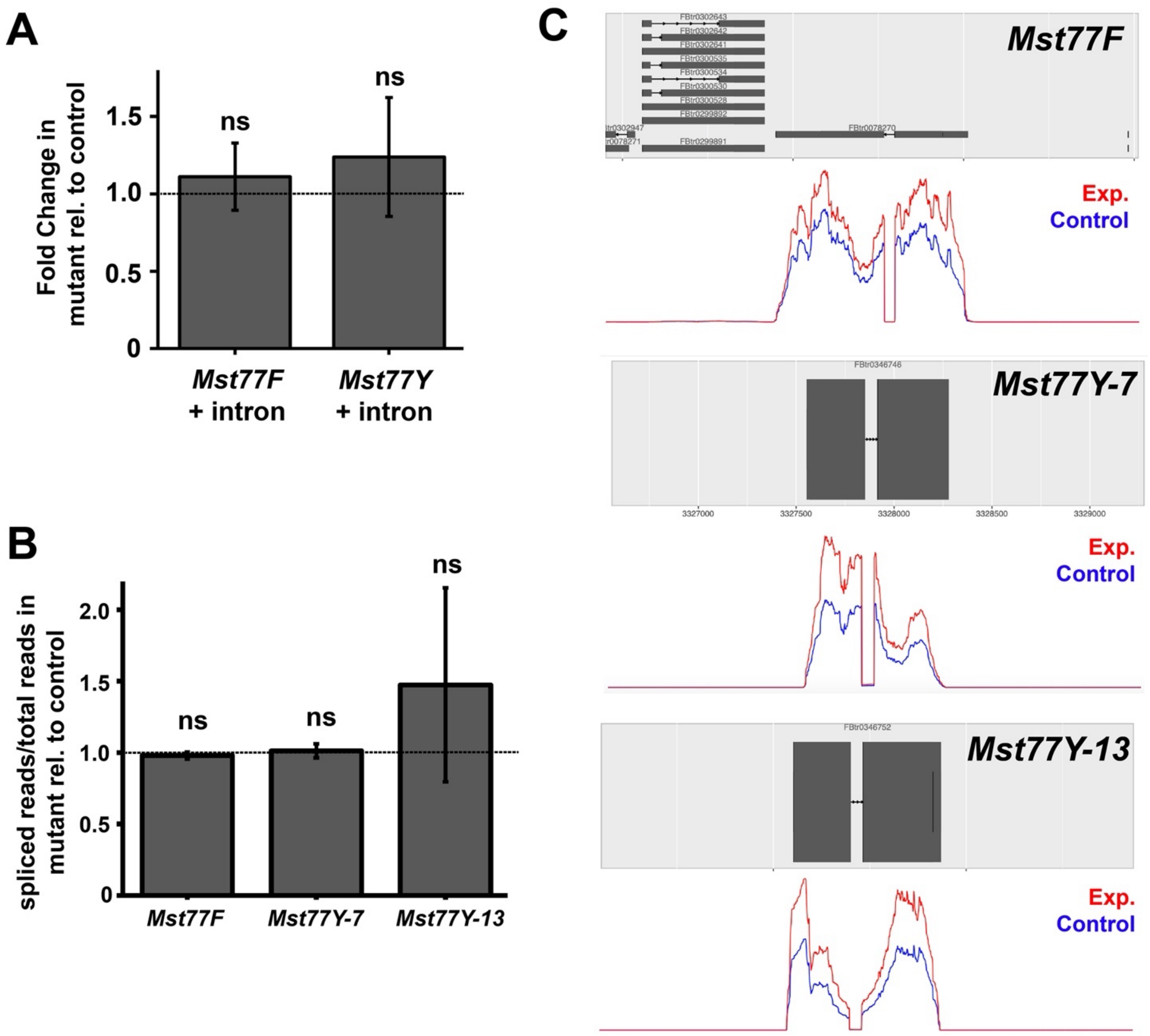

Fig. S10. ***modulo*** **mutant does not affect splicing of** ***Mst77F*** **and** ***Mst77Y*** **transcript.**
A) qRT-PCR using exon-intron primer sets to examine level of nascent intron-containing transcript of *Mst77F* and *Mst77Y* in mutant (*mod*^{L8}/*mod*^{07570}) compared to control (*mod*^{07570}/+). Primer sets provided in Fig. S11. Data was normalized to Rp49 and control. Mean ±SD from three technical replicates is shown. *P*-values calculated using unpaired Student's t-test assuming unequal variances with n = 6 replicates from 2 independent experiments. ns indicates *P* > 0.05. Similar results were obtained from two biological replicates. Primer locations shown in Fig. S11A.
B) splicing analysis using total RNA-seq reads comparing proportion of spliced reads to total reads in mutant (*mod*^{L8}/*mod*^{07570}) normalized to control (*mod*^{07570}/+). *P*-values calculated using unpaired Student's t-test assuming unequal variances with n = 3 independent experiments. ns indicates *P* > 0.05.
C) Scaled read coverage of *Mst77F*, *Mst77Y-7, Mst77Y-13* genes showing similar coverage between mutant (*mod*^{L8}/*mod*^{07570}) and control (*mod*^{07570}/+) of exons/introns *Mst77F* and *Mst77Y* genes.

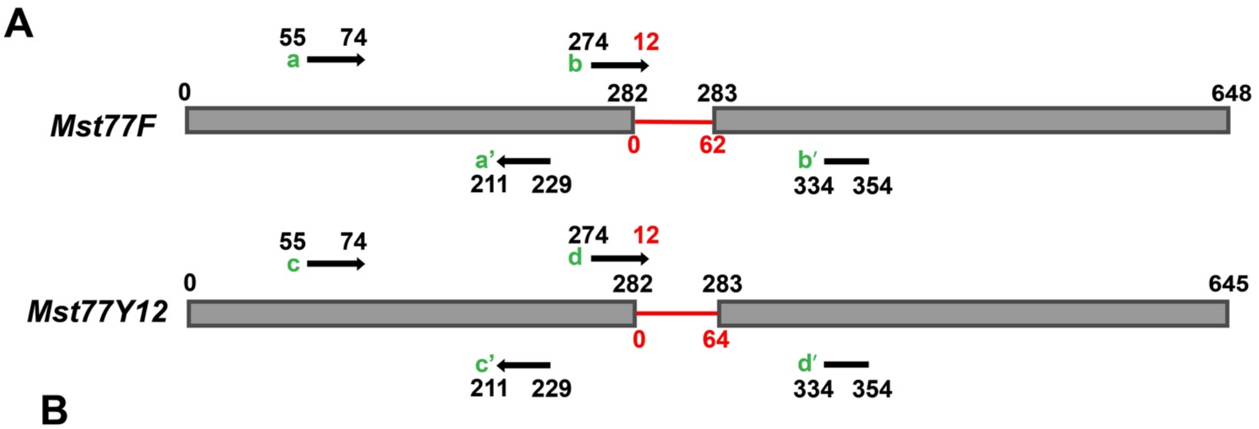

B

Primer ID	Sequence (5' to 3')
a (Mst77F_all_F)	GTGAAAACCTATAAAAAATC
a' (Mst77F_all_R)	CAGCCCGTCTTATCTCGTT
b (Mst77F_unspliced_F)	ATTCTGCGGTAAGTTAGGTG
b' (Mst77F_unspliced_R)	GTGCTCGCCCTCACTAGAAC
c (Mst77Y_all_F)	GTAAAAACCTCTAGAAAAGC
c' (Mst77Y_all_R)	CTGCCCGTCTTATCTGATA
d (Mst77Y_unspliced_F)	ACTCTGCGGTAA ATTATTGG
d' (Mst77Y_unspliced_R)	GAGCTCGGCCTCAATAGAGC

Fig. S11. qRT-PCR primers for *Mst77F/Mst77Y*

A) schematic of *Mst77F* and *Mst77Y* primer sets for qRT-PCR relative to location of exon junctions of respective genes.

B) table of respective primer sequences. Primers specific to *Mst77F* or *Mst77Y* could not be designed to span exon-exon junction due to high sequence homology in this region. Splicing changes were assessed by looking at relative amount of total transcript level vs. unspliced transcript level.

Supplementary Information

**Figure label**	***P*-value**
Fig. 1K	0
Fig. 2G	7.188×10^{-7}
Fig. 3E	7.34×10^{-4} (*UAS-Mst77Y-12*/+ vs. *β2-tub-Mst77Y-3*/+)<br>3.64×10^{-3} (*UAS-Mst77Y-12*/+ vs. *β2-tub-Mst77Y-12*/+)<br>0.750 (*β2-tub*-Mst77Y-3/+ vs. *β2-tub-Mst77Y-12*/+)
Fig. 3G	1.18×10^{-4}

Table S1. *P*-values in this study

β2-tub-**promoter** ***Mst77Y*** **SV40**
β2-tub-**Mst77Y-3-SV40**
TCATTGTAGGAGCCAGAGCCAATGGATCACCAAATCGTAGTTACAATCCTGTAGAGAACCATCCGCCGCCAAAATTTGGTTGTTAGACAAACCT<br>TCCTCCCTACGTAGATTTTTAAACCAGGATGGGTCATAATACATATAAGTTTGGAGAGCAAGGTTAATAGTCTTTAAAAGGCAGTTTTTGCTTAAG<br>AAATAATCGACCCATCCCATTATACACCCATATAAACATTTACAAAGGAGTAAAATCCAGGACATCCATGTCAATATCAATCGTATCATCTGGTCG<br>GTAGCCTTGGAATCCTCTATTGCTTCCAAGGCACCGCCAAATCCATCCCATCTCGAATTTTAGCCGTATATTCGTTTATCTATGTAAGTACTATTA<br>AAGTTTGTGCTCAAAACGGAGAACTGAGTTTTCTGAAATCGGGGTGTGTGAAATGTGTCGAAGTCGGAAATCGTAGTAGCCTATTTGTGAACAT<br>TCGGTGTAGTAATCCAAGCCAGGTTCAGTTCACCTCAGTATCAGCTAGCACGTACACGACTAAAATCTAAACCTGAAAAATTATACGTTTAAATA<br>TTCAGTCTTTTGCCGATTTTTGCCCCACTCAGACTGTTTTAAAAGCTCGATTTTTTTTTGTACCATTTTTTCGGTGTGAAAAAGGGGCCCTAACTT<br>TACTATCAAAATGAGCAATCTGAAACAAAAGGATATCAAGCCGGACGTGGCAGTTTCAAAATCAGTAAAAACCTCTAGAAAAGCAATTGAATATG<br>TTAAGTCCGACGCTTCCGACATTGACGAAGATATCAATAGGACAGAGTACGAATACGCCTCATCGTCTGGCTTTGTTAATTTTCTGAGGGACTTT<br>AAGAAACGTTATGGAGAATATTACTCGAATTATCAGATAAGACGGGCAGCTGAAACCCGATGGAACGAAATGTCATTCCGTCATCGATGCCAGT<br>ACTCTGCGGAACCATTGGACACTTTTCACGTAGAGCCGAACAGTGTGAGCAGTCTTCAGCGCTCTATTGAGGCCGAGCTCTGAAATAAGTGGC<br>TGCGACACTTTCTTTGGTGCCTGTGGCTCCAATAGCTGCACTCCAAGAAAGGAGAACAAGTGTTCCAAGCCCAGGGTCTGGAAGAGTTGCCCA<br>AAACCGCGGGCCAAGTCCTCGAAGCAACGTCGCAATTGCGCCAAACCGAAGCCCAAGTGCGCCCGACCCCGTACGGCATGTCCCCGCCCCA<br>GAAACAGTATGGAATGCGGCAAGCCGAAGGCAAAGCCAAGGTGTCTTAAGCCCAAGAGTTCCAAGCCCAAGTGCTCGGTGTAATGAAGGAAC<br>CTTACTTCTGTGGTGTGACATAATTGGACAAACTACCTACAGAGATTTAAAGCTCTAAGGTAAATATAAAATTTTTAAGTGTATAATGTGTTAAACT<br>ACTGATTCTAATTGTTTGTGTATTTTAGATTCCAACCTATGGAACTGATGAATGGGAGCAGTGGTGGAATGCCTTTAATGAGGAAAACCTGTTTT<br>GCTCAGAAGAAATGCCATCTAGTGATGATGAGGCTACTGCTGACTCTCAACATTCTACTCCTCCAAAAAAGAAGAGAAAGGTAGAAGACCCCAA<br>GGACTTTCCTTCAGAATTGCTAAGTTTTTTGAGTCATGCTGTGTTTAGTAATAGAACTCTTGCTTGCTTTGCTATTTACACCACAAAGGAAAAAGC<br>TGCACTGCTATACAAGAAAATTATGGAAAAATATTCTGTAACCTTTATAAGTAGGCATAACAGTTATAATCATAACATACTGTTTTTTCTTACTCCA<br>CACAGGCATAGAGTGTCTGCTATTAATAACTATGCTCAAAAATTGTGTACCTTTAGCTTTTTAATTTGTAAAGGGGTTAATAAGGAATATTTGATG<br>TATAGTGCCTTGACTAGAGATCATAATCAGCCATACCACATTTGTAGAGGTTTTACTTGCTTTAAAAAACCTCCCACACCTCCCCCTGAACCTGA<br>AACATAAAATGAATGCAATTGTTGTTGTTAACTTGTTTATTGCAGCTTATAATGGTTACAAATAAAGCAATAGCATCACAAATTTCACAAATAAAGC<br>ATTTTTTTCACTGCATTCTAGTTGTGGTTTGTCCAAACTCATCAATGTATCTTATCATGTCTGGATC
β2-tub-***Mst77Y-12*-SV40**
TCATTGTAGGAGCCAGAGCCAATGGATCACCAAATCGTAGTTACAATCCTGTAGAGAACCATCCGCCGCCAAAATTTGGTTGTTAGACAAACCT<br>TCCTCCCTACGTAGATTTTTAAACCAGGATGGGTCATAATACATATAAGTTTGGAGAGCAAGGTTAATAGTCTTTAAAAGGCAGTTTTTGCTTAAG<br>AAATAATCGACCCATCCCATTATACACCCATATAAACATTTACAAAGGAGTAAAATCCAGGACATCCATGTCAATATCAATCGTATCATCTGGTCG<br>GTAGCCTTGGAATCCTCTATTGCTTCCAAGGCACCGCCAAATCCATCCCATCTCGAATTTTAGCCGTATATTCGTTTATCTATGTAAGTACTATTA<br>AAGTTTGTGCTCAAAACGGAGAACTGAGTTTTCTGAAATCGGGGTGTGTGAAATGTGTCGAAGTCGGAAATCGTAGTAGCCTATTTGTGAACAT<br>TCGGTGTAGTAATCCAAGCCAGGTTCAGTTCACCTCAGTATCAGCTAGCACGTACACGACTAAAATCTAAACCTGAAAAATTATACGTTTAAATA<br>TTCAGTCTTTTGCCGATTTTTGCCCCACTCAGACTGTTTTAAAAGCTCGATTTTTTTTTGTACCATTTTTTCGGTGTGAAAAAGGGGCCCTAACTT<br>TACTATCAAAATGAGCAATCTGAAACAAAAGGATATCAAGCCGGACGTGGCAGTTTCAAAATCAGTAAAAACCTCTAGAAAAGCAATTGAATATG<br>TTAAGTCCGACGCTTCCGACATTGACGAAGATATCAATAGGACAGAGTACGAATACGCCTCATCGTCTGGCTTTGTTAATTTTCTGAGGGACTTT<br>AAGAAACGTTATGGAGAATATTACTCGAATTATCAGATAAGACGGGCAGCTGAAACCCGATGGAACGAAATGTCATTCCGTCATCGATGCCAGT<br>ACTCTGCGGAACCATTGGACACTTTTCACGTAGAGCCGAACAGAGTGAGCAGTCTTCAGCGCTCTATTGAGGCCGAGCTCAGAATTCACTCTG<br>AAATAAGTGGCTGCGACACTTTCTTTGGTGCCTGTGGCTCCAATAGCTGCACTCCAAGAAAGGAGAACAAGTGTTCCAAGCCCAGGGTCTGGA<br>AGAGTTGCCCAAAACCGCGGGCCAAGTCCTCGAAGCAACGTCGCAATTGCGCCAAACCGAAGCCCAAGTGCGCCCGACCCCGTAAGGCATGT<br>CCCCGCCCCAGAAACAGTATGGAATGCGGCAAGCCGAAGGCAAAGCCAAGGTGTCTTAAGCCCAAGAGTTCCAAGCCCAAGTGCTCGGTGTA<br>ATGAAGGAACCTTACTTCTGTGGTGTGACATAATTGGACAAACTACCTACAGAGATTTAAAGCTCTAAGGTAAATATAAAATTTTTAAGTGTATAA<br>TGTGTTAAACTACTGATTCTAATTGTTTGTGTATTTTAGATTCCAACCTATGGAACTGATGAATGGGAGCAGTGGTGGAATGCCTTTAATGAGGA<br>AAACCTGTTTTGCTCAGAAGAAATGCCATCTAGTGATGATGAGGCTACTGCTGACTCTCAACATTCTACTCCTCCAAAAAAGAAGAGAAAGGTA<br>GAAGACCCCAAGGACTTTCCTTCAGAATTGCTAAGTTTTTTGAGTCATGCTGTGTTTAGTAATAGAACTCTTGCTTGCTTTGCTATTTACACCACA<br>AAGGAAAAAGCTGCACTGCTATACAAGAAAATTATGGAAAAATATTCTGTAACCTTTATAAGTAGGCATAACAGTTATAATCATAACATACTGTTT<br>TTTCTTACTCCACACAGGCATAGAGTGTCTGCTATTAATAACTATGCTCAAAAATTGTGTACCTTTAGCTTTTTAATTTGTAAAGGGGTTAATAAG<br>GAATATTTGATGTATAGTGCCTTGACTAGAGATCATAATCAGCCATACCACATTTGTAGAGGTTTTACTTGCTTTAAAAAACCTCCCACACCTCCC<br>CCTGAACCTGAAACATAAAATGAATGCAATTGTTGTTGTTAACTTGTTTATTGCAGCTTATAATGGTTACAAATAAAGCAATAGCATCACAAATTT<br>CACAAATAAAGCATTTTTTTCACTGCATTCTAGTTGTGGTTTGTCCAAACTCATCAATGTATCTTATCATGTCTGGATC

Table S2. Mst77Y constructs utilized to generate transgenic Mst77Y overexpression lines
β2-tub promoter sequence (yellow), respective Mst77Y sequence (magenta), SV40 3’ UTR sequence (cyan)

```
3R:32052060..32054974
Stop codon  arm primer sites  gRNA PAM  Modulo Exon  Modulo intron/UTR

AATTCAGTGCGAACAAACCGATATATGTTTGGTCGTACCATAATCGCAGCCTATAATTAATATTTATTTTGCATTTTTGGCACCTATTTATATTTCCTAAGTGTGCCCTGGCCCATAgtaagtgac
ttttaataatttttctttcaataccattaacaattgcttgaattaacattaagtttcctactaagataaaatgcaactgcaggttgacttggctttttaatgaaatataatatatatattataaat
tcctttagAAACAGATGATTCATAGCTGATGATTGAGGAAACTTACCATTTGGAATGTATAAACACATGTAAAATCAGACCGTTGgtaaatatctaatcatagattgtatcctaagtcttaaataa
atactttatataaactgtttaatatttattttatttttaaagGCTTTTATTATTTTTATATGTATACATGTGCATGAACGGACTTTAAACCATATTTAGTAGTGGAATAGAGCTATAAAAATGAG
ATTGAAGTCTCTAAAATACTTTATTTAATCAGCCTTAGCACCGATAGAGCACAACGGTCACACTGTTCTGTCCCTCTTTTCCACGTGCATTTTATTGAACGAAACCACGCGTTGTTCAGTCCAAAC
TTGACTTTGCTAATTTTGCCCAGAAAACCTCTAAAAGCTGTCAAAATGGCCCAAAAGAAAGCCGTCACCGTCAAAGGAAAAAAAGCTACGAATGGAGAGGAAAAGCCTTTGGCCAAACGGGTAACC
AAGTCCACAAAGGTACAGGAAGAGGAGACAGTAGTCCCCCAATCGCCTTCCAAAAAATCCAGAAAACAGCCTGTTAAAGAAGTGCCGCAGTTCAGTGAAGAGGACGAGTCTGATGTGGAGGAGCAA
AATGACGAGCAGCCAGGGGACGACAGCGACTTTGAGgtaagctataaaaaaaaacgtggtcttctgaatggatatggctaacattacatgcatagtatgcacgtatatatagatctgcatggataag
aatccattttacgttttctttgccacgtggttgtaaagattttataattataagacgagtgcacataacccacgatcctctggttttataccgactcggttcatatatcgataaaaaaaaaattgt
ctttattggtgctctcccatcagcggttataagaaaattgtctacttttcagACCGAAGAGGCAGCGGGCTTAATTGACGACGAGGCTGAGGAAGATGAAGAGTACAATAGTGACGATGAGGAAGA
TGATGATGACGATGAGTTGGAGCCTGGTGAGGTTTCCAAAAGTGAAGGCGCGGATGAGGTAGATGAATCCGATGATGATGAAGAAGCGCCTGTAGAGAAGCCAGTTTCCAAGAAATCAGAAAAAGC
CAACTCTGAAAAGTCCGAAGAAAATAGAGGAATTCCAAAAGTAAAGGTTGGCAAGATTCCGCTGGGAACGCCAAAAAACCAGATCGTTTTTGTCACAAACCTACCAAACGgtaaagtaactggttt
attatttcatttttcaagtatatttaaacttagtatatttgttccacatagAGTATCTCCACAAGGACTTGGTCGCATTGTTCGCCAAGTTCGGACGACTTTCCGCTTTGCAGCGTTTCACTAATT
TGAATGGCAATAAATCAGTTCTCATTGCCTTTGATACGTCAACTGGAGCAGAAGCTGTTCTGCAGGCTAAGCCTAAAGCGTTAACCCTTGGAGACAATGTTTTGTCTGTGTCCCAACCGAGAAATA
AGGAAGAGAACAACGAACGCACGGTGGTGGTTGGCCTGATTGGACCTAATATTACCAAGGACGACTTAAAAACCTTTTTTGAGAAGGTAGCTCCCGTGGAGGCGGTGACTATATCCAGTAACCGCC
TCATGCCCAGAGCTTTTGTACGTTTGGCGTCGGTCGATGACATACCGAAGGCTCTTAAACTGCATAGCACTGAGCTGTTTTCTCGCTTTATAACGGTTCGTCGGATCTCACAAGAATCGATTTCAC
GTACCAGTGAACTTACCTTGGTTGTCGAAAATGTGGGCAAACACGAGTCTTACAGCTCCGACGCTCTAGAAAAGATATTCAAAAAATTTGGTGATGTTGAAGAAATCGACGTTGTGTGCAGCAAGG
CAGTTTTAGCTTTTGTCACGTTCAAGCAGTCGGATGCCGCCACGAAGGCTTTAGCACAACTCGACGGAAAGACTGTTAATAAGTTTGAGTGGAAGCTTCACCGATTCGAACGCAGCACCTCGGGGA
GGGCCATTCTTGTGACGAACTTGACTTCAGgtgggttacccgtttcgtaaaattgtttaaaattattaatcattatcatgtaaaatttcgttgtagATGCTACTGAAGCCGACCTGCGGAAAGTGT
TTAACGATAGCGGCGAAATCGAAAGCATCATAATGTTGGGCCAAAAAGCGGTAGTAAAGTTCAAGGACGATGAGGGTTTCTGCAAATCTTTCTTGGCTAATGAAAGCATTGTAAACAACGCCCCTA
TATTTATTGAGCCAAACTCTCTGCTGAAGCATAGATTGTTAAAGAAACGCTTAGCTATTGGTCAGACCCGCGCCCCGCGGAAGTTCCAAAAGGACACTAAGCCAAACTTTGGTAAAAAACCATTTA
ACAAGCGCCCGGCACAAGAGAATGGTGGTAAATCGTTTGTTAAAAGGGCAAGATTTTAGAACTTGAACACTACAAGAATTAGCTTTAAGTTGCGTAGTACCATAAATACGGCCACAGCCCCGATAA
TTCCATACAACTTGATGTTATGGATTTAGTTGATAAGAGATACTCCATTAAATTTTTACCGGAGAAATGCTAGCCGTATTCAAGCAGATGTCGATTTTTATAAGCATCGGACATGGAACTAAATAA
AAGTAAATAAAAACCCCGATAAGAATTTTGTATTCAACGTCTTGGTTTTGGGTAACTAGATACTTCAAAAAACGATTTACTGCTTATATGAACGTATACAGGTGCGATCTCCAGTATATTCCGTTT
TTTTTTTGTGTTTGAATTTTATCTCGCTTCGGTACTCTCGTATATAGGTTCCTATCCCTTCCAAATGGCCCAATGTACAGCTGCGCTTGTTCAAAAAGCGAGAACTATATGAATTTCCTTTGTAT
AATTAATTTATTAGTAATACATTTTTGTAGTATTTTATAATTTTTTGAATTTTTTTGTTCATACTCAGAAATCTAATATGGTTGGCTAACATATGTATAAACTTGTAAATGTTTGTATGTATGTTT
ATATTTTTTGTAAATGCCATTTAGATTAGTACTTTAAATTTCAGTAATTGTTGGTTCAAAAAATTAGTTTATCCTTTTTATGTGTAAATATGTATAAAAAATCATCACTTGGATAAAAGCAACGTA
TAGGGAGTTTAGCCAACCTAAAAAAAACCCTTTCGATTAGGAATTTCCCAATCACTCTGTTTATATATAGTTCGTTCAAGCTACAAAAAACTGTGCTCGTGACGCGTTTGGCTTACTGACAAGTTT
TTGTGTGTTATATGTAAGTCTCTGTCTCATGTTGCTTTTGACCTTGTTTTATAAGAAATATAATATCGCTATCGTAATATAGCTTAGGCAGAAAATCACTAATAAGGCAAATTAAAGTGAAGGGCA
CTGTGTGGGACTTCTGAAAATACATAATGCAAACAAGCTTACTATTTAAACTTGACAACTTATATATCTCAGAGTTCGTTTAAGCCGTGCATAACTCATATACGTTGTATAGGCAAATTATAAGCC
CTAGAATATTACTTTTTTTAGATATACTTTCACGAGAGCTTTTTCTTTCATGACGAATTGAAGTAGGTGACACTATCTTTCACAAAGCTTATAAAATTATGCCTACACAAAGGATAAAAGAATACA
AAAATAAAACAACTGAAAGATTTTTAAAATATTTCTTGTTATTTTTATATAATTGTAGTATTGTATTAAACAGACAAATAAAGAGCGTATAAGTGTGCTGGAGATACATTCAGTTTTTTGATCCAT
GTGGCATAAACAACAAATTATAAATAATAATACATTTTTAACTGAGTCACAAAAAAATATAAGTCAAACGAAATAAAATATGAAATGGAAAACGTTTTAAAATTATAAGAAAACACTGGGTAAAAG
CT
```

Arm Primer	Sequence (5′-3′)
2050KI 5	CCTCTTCGCTATTACGCCAGGTGGAGGCGGTGACTATA
2050KI 3	GCTATGACCATGATTACGCCAGCAACATGAGACAGAGACT

gRNA	Target
2050KI-sg1	AAGCGCCCGGCACAAGAGAATGG
2050KI-sg2	GCGCCCGGCACAAGAGAATGGTGG

```
Modulo-gfp insert
Linker  gRNA PAM  donor point-mutation  GFP  Stop codon  Modulo Exon  Modulo intron

GTGGAGGCGGTGACTATATCCAGTAACCGCCTCATGCCCAGAGCTTTTGTACGTTTGGCGTCGGTCGATGACATACCGAAGGCTCTTAAACTGCATAGCACTGAGCTGTTTTCTCGCTTTATAACG
GTTCGTCGGATCTCACAAGAATCGATTTCACGTACCAGTGAACTTACCTTGGTTGTCGAAAATGTGGGCAAACACGAGTCTTACAGCTCCGACGCTCTAGAAAAGATATTCAAAAAATTTGGTGAT
GTTGAAGAAATCGACGTTGTGTGCAGCAAGGCAGTTTTAGCTTTTGTCACGTTCAAGCAGTCGGATGCCGCCACGAAGGCTTTAGCACAACTCGACGGAAAGACTGTTAATAAGTTTGAGTGGAAG
CTTCACCGATTCGAACGCAGCACCTCGGGGAGGGCCATTCTTGTGACGAACTTGACTTCAGgtgggttacccgtttcgtaaaattgtttaaaattattaatcattatcatgtaaaatttcgttgta
gATGCTACTGAAGCCGACCTGCGGAAAGTGTTTAACGATAGCGGCGAAATCGAAAGCATCATAATGTTGGGCCAAAAAGCGGTAGTAAAGTTCAAGGACGATGAGGGTTTCTGCAAATCTTTCTTG
GCTAATGAAAGCATTGTAAACAACGCCCCTATATTTATTGAGCCAAACTCTCTGCTGAAGCATAGATTGTTAAAGAAACGCTTAGCTATTGGTCAGACCCGCGCCCCGCGGAAGTTCCAAAAGGAC
ACTAAGCCAAACTTTGGTAAAAAACCATTTAACAAGCGCCCGGCACAAGAAAACGGTGGTAAATCGTTTGTTAAAAGGGCAAGATTTggaggtGtgagcaagggcgaggagctgttcaccggggtg
gtgcccatcctggtcgagctggacggcgacgtaaacggccacaagttcagcgtgtccggcgagggcgagggcgatgccacctacggcaagctgaccctgaagttcatctgcaccaccggcaagctg
cccgtgccctggcccaccctcgtgaccaccctgacctacggcgtgcagtgcttcagccgctaccccgaccacatgaagcagcacgacttcttcaagtccgccatgcccgaaggctacgtccaggag
cgcaccatcttcttcaaggacgacggcaactacaagacccgcgccgaggtgaagttcgagggcgacaccctggtgaaccgcatcgagctgaagggcatcgacttcaaggaggacggcaacatcctg
gggcacaagctggagtacaactacaacagccacaacgtctatatcatggccgacaagcagaagaacggcatcaaggtgaacttcaagatccgccacaacatcgaggacggcagcgtgcagctcgcc
gaccactaccagcagaacacccccatcggcgacggccccgtgctgctgcccgacaaccactacctgagcacccagtccgccctgagcaaagaccccaacgagaagcgcgatcacatggtcctgctg
gagttcgtgaccgccgccgggatcactctcggcatggacgagctgtacaagTAGAACTTGAACACTACAAGAATTAGCTTTAAGTTGCGTAGTACCATAAATACGGCCACAGCCCCGATAATTCCA
TACAACTTGATGTTATGGATTTAGTTGATAAGAGATACTCCATTAAATTTTTACCGGAGAAATGCTAGCCGTATTCAAGCAGATGTCGATTTTTATAAGCATCGGACATGGAACTAAATAAAAGTA
AATAAAAACCCCGATAAGAATTTTGTATTCAACGTCTTGGTTTTGGGTAACTAGATACTTCAAAAAACGATTTACTGCTTATATGAACGTATACAGGTGCGATCTCCAGTATATTCCGTTTTTTTT
TTTGTGTTTGAATTTTATCTCGCTTCGGTACTCTCGTATATAGGTTCCTATCCCTTCCAAATGGCCCAATGTACAGCTGCGCTTGTTCAAAAAGCGAGAACTATATGAATTTCCTTTGTATAATTA
ATTTATTAGTAATACATTTTTGTAGTATTTTATAATTTTTTGAATTTTTTTGTTCATACTCAGAAATCTAATATGGTTGGCTAACATATGTATAAACTTGTAAATGTTTGTATGTATGTTTATATT
TTTTGTAAATGCCATTTAGATTAGTACTTTAAATTTCAGTAATTGTTGGTTCAAAAAATTAGTTTATCCTTTTTATGTGTAAATATGTATAAAAAATCATCACTTGGATAAAAGCAACGTATAGGG
AGTTTAGCCAACCTAAAAAAAACCCTTTCGATTAGGAATTTCCCAATCACTCTGTTTATATATAGTTCGTTCAAGCTACAAAAAACTGTGCTCGTGACGCGTTTGGCTTACTGACAAGTTTTTGTG
TGTTATATGTAAGTCTCTGTCTCATGTTGC
```

Table S3. CRISPR/Cas9 generation of *modulo-gfp* at endogenous locus

FISH Probe recognizing:	Sequences (5' to 3')
**RNA probes:**	
Poly(A)	Cy5-TTTTTTTTTTTTTTTTTTTTTTTTTTTTTT<br>Cy3-TTTTTTTTTTTTTTTTTTTTTTTTTTTTTT
Mst77F	Custom Stellaris® probe set (Quasar 570)<br>1. GATTTTGTTACTGCCACCTC<br>2. TTGATTTTTTATAGGTTTTT<br>3. ATTCTCCGTAGCGCTTCTTA<br>4. CGTCTTATCTCGTTATTCG<br>5. TGTGCTCGCCCTCACTAGAACG<br>6. CGAGGTTTTCGCCCGTGGTT<br>7. CATTCCATCTTCTTCCTGGG
Mst77Y	Custom Stellaris® probe set (Quasar 670)<br>1. GATTTTGAAACTGCCACGTC<br>2. TTGCTTTTCTAGAGGTTTTT<br>3. ATTCTCCATAACGTTTCTTA<br>4. CGTCTTATCTGATAATTCG<br>5. TGAGCTCGGCCTCAATAGAGCG<br>6. CGAGGACTTGGCCCGCGGTT<br>7. CATTCCATACTGTTTCTGGG
**DNA probes:**	
X-chromosome satellite: $(TAGA)_n$	$(TAGA)_8$-Cy3
Y-chromosome satellite: $(AATAC)_n$	$(AATAC)_6$-Cy5

Table S4. FISH probes utilized in this study
